# Comparison of 3 Different Minimally Invasive Surgical Techniques for Lumbar Spinal Stenosis: A Randomized Clinical Trial

**DOI:** 10.1001/jamanetworkopen.2022.4291

**Published:** 2022-03-28

**Authors:** Erland Hermansen, Ivar Magne Austevoll, Christian Hellum, Kjersti Storheim, Tor Åge Myklebust, Jørn Aaen, Hasan Banitalebi, Masoud Anvar, Frode Rekeland, Jens Ivar Brox, Eric Franssen, Clemens Weber, Tore K. Solberg, Håvard Furunes, Oliver Grundnes, Helena Brisby, Kari Indrekvam

**Affiliations:** 1 Department of Orthopedic Surgery, Ålesund Hospital, Møre and Romsdal Hospital Trust, Ålesund, Norway; 2 Kysthospitalet in Hagevik, Orthopedic Clinic, Haukeland University Hospital, Bergen, Norway; 3 Department of Clinical Medicine, University of Bergen, Bergen, Norway; 4 Hofseth BioCare, Ålesund, Norway; 5 Division of Orthopedic Surgery, Oslo University Hospital Ullevål, Oslo, Norway; 6 Communication and Research Unit for Musculoskeletal Health (FORMI), Oslo University Hospital, Oslo, Norway; 7 Department of Research and Innovation, Møre and Romsdal Hospital Trust, Ålesund, Norway; 8 Department of Registration, Cancer Registry Norway, Oslo, Norway; 9 Department of Circulation and Medical Imaging, Faculty of Medicine and Health Sciences, Norwegian University of Science and Technology, Trondheim, Norway; 10 Department of Diagnostic Imaging, Akershus University Hospital, Oslo, Norway; 11 Institute of Clinical Medicine, University of Oslo, Oslo, Norway; 12 Unilabs Radiology, Oslo, Norway; 13 Department of Physical Medicine and Rehabilitation, Oslo University Hospital, Oslo, Norway; 14 Department of Orthopedics, Stavanger University Hospital, Stavanger, Norway; 15 Department of Neurosurgery, Stavanger University Hospital, Stavanger, Norway; 16 Department of Quality and Health Technology, University of Stavanger, Stavanger, Norway; 17 Department of Neurosurgery and the Norwegian Registry for Spine Surgery, University Hospital of Northern Norway, Tromsø, Norway; 18 Institute of Clinical Medicine, The Arctic University of Norway, Tromsø, Norway; 19 Department of Surgery, Gjøvik Hospital, Innlandet Hospital Trust, Brumunddal, Norway; 20 Institute of Health and Society Studies, University of Oslo, Oslo, Norway; 21 Department of Orthopedics, Akershus University Hospital, Oslo, Norway; 22 Department of Orthopedics, Sahlgrenska University Hospital, Gothenburg, Sweden; 23 Department of Orthopedics, Institute for Clinical Sciences, Sahlgrenska Academy, University of Gothenburg, Gothenburg, Sweden

## Abstract

**Importance:**

Operations for lumbar spinal stenosis is the most often performed surgical procedure in the adult lumbar spine. This study reports the clinical outcome of the 3 most commonly used minimally invasive posterior decompression techniques.

**Objective:**

To compare the effectiveness of 3 minimally invasive posterior decompression techniques for lumbar spinal stenosis.

**Design, Setting, and Participants:**

This randomized clinical trial used a parallel group design and included patients with symptomatic and radiologically verified lumbar spinal stenosis without degenerative spondylolisthesis. Patients were enrolled between February 2014 and October 2018 at the orthopedic and neurosurgical departments of 16 Norwegian public hospitals. Statistical analysis was performed in the period from May to June 2021.

**Interventions:**

Patients were randomized to undergo 1 of the 3 minimally invasive posterior decompression techniques: unilateral laminotomy with crossover, bilateral laminotomy, and spinous process osteotomy.

**Main Outcomes and Measures:**

Primary outcome was change in disability measured with Oswestry Disability Index (ODI; range 0-100), presented as mean change from baseline to 2-year follow-up and proportions of patients classified as success (>30% reduction in ODI). Secondary outcomes were mean change in quality of life, disease-specific symptom severity measured with Zurich Claudication Questionnaire (ZCQ), back pain and leg pain on a 10-point numeric rating score (NRS), patient perceived benefit of the surgical procedure, duration of the surgical procedure, blood loss, perioperative complications, number of reoperations, and length of hospital stay.

**Results:**

In total, 437 patients were included with a median (IQR) age of 68 (62-73) years and 230 men (53%). Of the included patients, 146 were randomized to unilateral laminotomy with crossover, 142 to bilateral laminotomy, and 149 to spinous process osteotomy. The unilateral laminotomy with crossover group had a mean change of −17.9 ODI points (95% CI, −20.8 to −14.9), the bilateral laminotomy group had a mean change of −19.7 ODI points (95% CI, −22.7 to −16.8), and the spinous process osteotomy group had a mean change of –19.9 ODI points (95% CI, −22.8 to –17.0). There were no significant differences in primary or secondary outcomes among the 3 surgical procedures, except a longer duration of the surgical procedure in the bilateral laminotomy group.

**Conclusions and Relevance:**

No differences in clinical outcomes or complication rates were found among the 3 minimally invasive posterior decompression techniques used to treat patients with lumbar spinal stenosis.

**Trial Registration:**

ClinicalTrials.gov Identifier: NCT02007083

## Key Points

**Question:**

Are any of the 3 most common minimally invasive procedures for surgical treatment of lumbar spinal stenosis superior to the others?

**Findings:**

In this randomized clinical trial of 437 patients at 16 public hospitals in Norway, there was no difference in the clinical results from the 3 most commonly performed minimally invasive decompression techniques investigated after 2 years.

**Meaning:**

These findings suggest that surgeons can choose decompression techniques according to their skills and preferences.

## Teaser

This randomized clinical trial compares the effectiveness of 3 minimally invasive posterior decompression techniques for lumbar spinal stenosis at 16 public hospitals in Norway.

## Introduction

Symptomatic lumbar spinal stenosis (LSS) is characterized by pain and discomfort in the lower back and the lower extremities, impaired walking ability, and functional disability. Imaging shows a narrowing of the lumbar spinal canal.^1^ LSS is a common condition involving a large patient group who are treated by several medical specialties involved in different aspects of the diagnosis and treatments. Several studies^2,3,4,5^ have shown superior clinical results after surgical treatment compared with nonsurgical treatment. The surgical procedure for LSS is the most frequently performed procedure in the adult lumbar spine.^6,7^

A posterior decompression at the level of the stenosis is usually performed, and an open laminectomy has been considered the reference standard.^8,9^ Less invasive, midline retaining, posterior decompression techniques have been introduced in the last decades. They have shown similar effectiveness as traditional laminectomies, but the duration of the surgical procedure and length of hospital stay is usually shorter because of the less invasive nature of the procedure.^10^

Various midline retaining techniques have been introduced with scarce scientific evidence regarding the possible advantages and disadvantages. Therefore an effectiveness study of different minimally invasive techniques would be of interest for the medical community and health care planning and allocation of resources. This trial investigates the outcome after 3 commonly used methods that differ in spinal canal access and may differ in surgical radicality.

Some former trials^11,12,13,14,15,16,17^ show comparable clinical results after different posterior decompression techniques. However, 2 Cochrane reviews, Overdevest et al^8^ and Machado et al,^18^ and an umbrella review by Jacobs et al^19^ concluded that the scientific evidence is of low quality and that high-quality research is required before a scientific conclusion can be reached. This trial aims to investigate whether 1 of the 3 most commonly used minimally invasive posterior decompression techniques is superior in the treatment of LSS with respect to clinical outcomes.

## Methods

The Norwegian Degenerative Spondylolisthesis and Spinal Stenosis study (NORDSTEN study) consists of 2 randomized clinical trials, including the NORDSTEN Spinal Stenosis Trial (SST). The SST is a multicenter trial where orthopedic and neurosurgical departments of 16 hospitals participated. The study protocol was prepared according to the Standard Protocol Items: Recommendations for Interventional Trials (SPIRIT) reporting guideline.^20^ The protocol is also attached in Supplement 1. The trial was registered at ClinicalTrials.gov (NCT02007083). Ethics approval was given by the Regional Committee for Medical and Health Research Ethics of Central Norway. This randomized clinical trial is reported according to the Consolidated Standards of Reporting Trials (CONSORT) reporting guideline.^21^ The trial was also monitored according to a modified version of the International Conference on Harmonization Guideline for Good Clinical Practice (ICH-GCP),^22^ and a monitor-report is provided in eAppendix 1 in Supplement 2. A patient representative from the Norwegian Back Association has been a permanent member of both the Scientific Steering Committee and the Working Committee of the NORDSTEN-study (eAppendix 2 in Supplement 2). Informed consent was collected in a paper-based form and was stored in a fire proof safe at each of the study centers according to Norwegian rules for conducting clinical trials.

### Inclusion Process and Patient Recruitment

Patients with symptoms of LSS and corresponding magnetic resonance imaging findings were eligible for inclusion in the trial. An orthopedic or neurosurgical surgeon assessed the participants at 1 of the 16 participating hospitals between February 2014 and October 2018. Patients with degenerative spondylolisthesis were excluded. Eligibility criteria are presented in the eTable 1 in Supplement 2. Initially, patients were excluded from February 2014 to October 2015 if their Oswestry Disability Index (ODI) at baseline was less than 25 points. The removal of this exclusion criterion was done to increase the external pragmatism and validity of the study. An amendment was sent to the ethics authorities, and the amendment was also registered in ClinicalTrials.gov and in the published protocol.^20^

### Randomization and Masking

Patients who provided informed consent were randomized to 1 of the 3 different posterior decompression techniques. The randomization (ie, 1:1:1 allocation) was performed within 6 weeks before the surgical procedure. We used a block randomization design, stratified by 16 hospitals, with the blocks being made as small as possible (randomly selected block size 3 and 6) to ensure that every hospital performed similar amount of all 3 procedures. The randomization procedure was concealed (computer-generated) and administered by the NORDSTEN-study coordination center located at the Communication and Research Unit for Musculoskeletal Health, Oslo University Hospital, Oslo, Norway. Output information regarding allocation was emailed to the local study coordinator, who was not involved in the recruitment or treatment of patients and registered in the patient record. The patients were not blinded to the treatment group; they were informed that none of the treatment options were documented as superior to the other.

### Surgical Techniques

**Figure 1.**
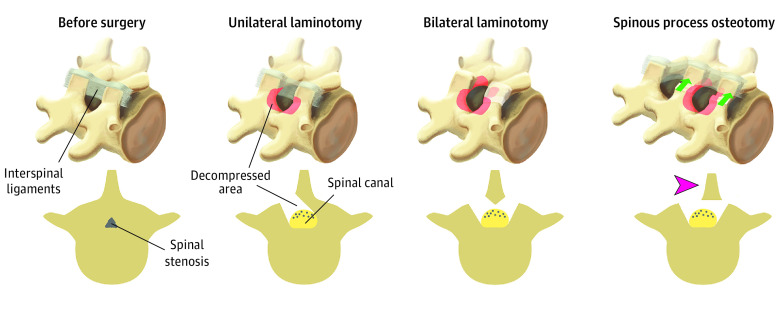
Before and After the Surgical Procedure for Lumbar Spinal Stenosis With the 3 Different Minimally Invasive Decompression Techniques Used in the Study

All surgeons were familiar with the 3 techniques through previous experience, the surgical protocol, and joint demonstration operations were performed before initiating the study (Figure 1). The surgical target level was confirmed by intraoperative fluoroscopic guidance. When performing unilateral laminotomy with crossover (UL), loupe magnification, or surgical microscope was mandatory, while in bilateral laminotomy (BL) and spinous process osteotomy (SPO) the use of loupes or microscope was optional depending on the surgeon’s preference. The surgeons were instructed to visualize the respective medial borders of the pedicles and the nerve roots from the beginning of the thecal sac passing the pedicle.

### UL With Crossover

An ipsilateral flavectomy was performed followed by a laminotomy of the lower part of the superior lamina and the upper part of the inferior lamina.^23^ Laterally, a medial facetectomy was performed, and the patient was then slightly rotated to visualize the contralateral side. The dura was retracted, and the decompression was performed contralaterally.

### BL

A bilateral flavectomy was performed followed by a bilateral laminotomy of the lower part of the superior lamina and the upper part of the inferior lamina. Laterally, a medial facetectomy was performed.^24^

### SPO and Decompression

An osteotomy was performed at the base of the spinous process above and sometimes below the affected level.^25^ The spinous process was retracted to the contralateral side with intact supraspinal and interspinal ligaments to access and decompress the spinal canal in the midline. A laminotomy of the lower part of the superior lamina and the upper part of the inferior lamina was performed followed by a medial facetectomy. Both nerve roots were visualized, and the lateral recesses were decompressed. Special attention was warranted when a multilevel decompression was performed to preserve at least one-third of the lamina.

### Primary Outcome

The primary outcome measure was change in disability measured with Oswestry Disability Index (ODI, version 2.0) from baseline to 24 months after the surgical procedure. ODI is a widely used and validated pain and function score, where 0 is considered asymptomatic and 100 is considered completely disabled.^26,27^ The patients completed the questionnaires, including ODI, before the surgical procedure and at 3, 12, and 24 months after the surgical procedure. The mean score change from baseline to 2-year follow-up was compared between the 3 groups. Additionally, patient outcomes were classified as a success if they had a 30% reduction of baseline ODI, and the proportion of patients classified as a success in each group were determined at the different follow-up time points.^28,29^

### Secondary Outcomes

Secondary patient-reported outcomes were changes from baseline to follow-up in the EuroQol 5-dimensional questionnaire utility index (EQ-5D), the Zurich Claudication Questionnaire (ZCQ-score), a 10-point numeric rating scale (NRS) for low back pain and leg pain, and a global perceived effect (GPE)-scale.

The EQ-5D is a generic quality-of-life questionnaire, ranging from −0.59 (ie, worst possible) to 1.00 (ie, best possible). This questionnaire was validated for the Norwegian population.^30^ The 3-level version of EQ-5D and the corresponding UK value set to calculate scores was used.

The ZCQ is a disease-specific questionnaire for LSS and includes symptom severity, physical activity, and patient satisfaction during follow-up. Answers range from 1.0 to 5.0 in the symptom severity scale.^31^ In the physical activity scale, the range is from 1.0 to 4.0. The patient satisfaction scale was answered postoperatively and ranged from 1.0 to 4.0. For all scales, 1.0 is the best option.

The NRS scores for leg and low back pain are validated parameters for clinical trials.^32^ The range is from 0 to 10, where 0 is no pain, and 10 is the worst pain imaginable.

The global perceived effect (GPE) scale is a 7-point score, which is recommended for clinical trials of chronic pain conditions.^33^ It has 7 response categories: 1, completely recovered; 2, much improved; 3, slightly improved; 4, no change; 5, slightly worse; 6, much worse; 7, worse than ever.

Surgical data defined as secondary outcomes were duration of the procedure, perioperative bleeding volume, complications, number of reoperations, and length of hospital stay. All primary and secondary outcomes data were administered by paper-based patient-reported questionnaires and case report form. The data were obtained from the patient and an independent study coordinator at each hospital, and the data was registered electronically by the study coordinating center.

### Statistical Analysis

The trial was conducted with a superiority design to detect a difference in mean change of ODI from baseline to 2-year follow-up of 7 points between any groups. Because this requires 3 tests, the significance level was lowered from the standard *P* = .05 to *P* = .02. With an assumed SD of 18, 80% power, and a dropout rate of 15%, the sample size estimation recommended 155 patients in each group and 465 total. However, inclusion was stopped after reaching 437 patients because of the low rate of inclusion in the last months of the inclusion period. At that time, we had reports that the dropout rate was lower than anticipated, ensuring a sufficient number of participants in the statistical analysis.

Standard descriptive statistics were presented as absolute and relative frequencies for categorical variables, as mean (SD) for continuous variables, and as median (IQR) if skewed. Normal distribution was determined by visual inspection of histograms and qq-plots. Outcomes were analyzed by estimating multilevel linear models, including a random intercept for operating hospitals to account for the multicenter design. Continuous outcomes were analyzed using multilevel linear regressions, adjusting for baseline measurement if the outcome was a change score, and proportions were analyzed using multilevel Poisson regressions and adjusted for baseline measurement where appropriate. Predicted marginal effects, with corresponding 95% CIs were presented for all outcomes for all study arms. Actual means with corresponding 95% CIs were also presented graphically, along with standard *t* tests, as outlined in a published study protocol. Potential interaction effects were analyzed by including interaction terms between study arms and the variable of interest, tested using likelihood ratio tests, and comapred models with and without interaction effects. Complete case analyses were prefered because the proportion of missing observations rarely exceeded 10%. To assess the robustness of the results, we also analyzed the primary outcome after imputing missing data using multiple imputations with chained equations, including study arm, patients’ age, sex, body mass index (BMI; calculated as weight in kilograms divided by height in meters squared), and smoking status as factors in the imputation models.

Analyses were performed following the intention-to-treat principle (ITT). As 386 of 393 (98%) of the study participants that were eligible for analyses (ie, had necessary measurements of ODI at baseline and 2-year follow-up), were treated according to randomization, per-protocol analyses were deemed unnecessary. All analyses were executed by a statistician (T.Å.M.) blinded to the treatment given. Statistical analyses were performed using Stata Statistical Software version 17 (StataCorp). Two-sided *t* tests were used for the calculation of *P* values, and statistical significance was set at *P* = .02 for the primary outcome. Statistical analyses were performed in the period from May to June 2021.

## Results

### Baseline Data

Baseline characteristics are given in Table 1. Median (IQR) age in the total cohort was 68 (62-73) years with a mean (SD) BMI of 27.8 (4.2) and included 230 men (53%) and 87 individuals (21%) who smoked. The mean (SD) pain and function scores at baseline for the whole cohort were ODI, 38.4 (14.5); EQ-5D, 0.38 (0.32); ZCQ symptoms, 3.4 (0.6); ZCQ function, 2.5 (0.5); NRS leg pain, 6.5 (2.0); and NRS low back pain, 6.3 (2.2).

**Table 1.** Postrandomization Baseline Characteristics, Patient-Reported Outcome Measures, and Number of Levels of the Patients Included in the 3 Study Groups

Characteristic	No./total no. (%)		
	UL (n = 146)	BL (n = 142)	SPO (n = 149)
Age, median (IQR), y	69 (64-74)	67 (60-74)	68 (61-72)
Sex			
Female	73/146 (50.0)	78/142 (54.9)	55/149 (36.9)
Male	73/146 (50.0)	63/142 (45.1)	94/149 (63.1)
Higher level of education^a^	45/138 (32.6)	35/138 (25.4)	39/141 (27.7)
Smoking	23/138 (16.7)	34/139 (24.5)	30/141 (21.3)
BMI, mean (SD)	28.1 (4.2)	27.7 (3.9)	27.5 (4.4)
Former surgical procedure	11/133 (8.3)	10/133 (7.5)	8/134 (6.0)
Duration of leg pain >1 y	92/135 (68.2)	94/131 (71.8)	88/130 (67.7)
Duration of back pain >1 y	109/134 (81.3)	107/136 (78.7)	105/139 (75.5)
Use of analgesics	24/139 (17.3)	36/138 (26.1)	48/137 (35.0)
ASA score			
1	11/137 (8.0)	26/137 (19.0)	12/137 (8.8)
2	96/137 (70.1)	86/137 (62.8)	98/137 (71.5)
3	30/137 (21.9)	25/137 (18.3)	27/137 (19.7)
HSCL-25, median (IQR)	1.5 (1.2-1.9)	1.6 (1.3-1.9)	1.5 (1.3-1.8)
ODI, mean (SD)^b^	38.5 (14.9)	40.2 (14.1)	36.6 (14.3)
ZCQ, mean (SD)			
Symptom severity	3.4 (0.5)	3.4 (0.6)	3.3 (0.5)
Physical activity	2.5 (0.5)	2.6 (0.5)	2.5 (0.5)
NRS, median (IQR)^c^			
Leg pain	7 (5-8)	7 (5-8)	7 (5-8)
Back pain	7 (5-8)	7 (5-8)	7 (5-8)
EQ-5D, mean (SD)	0.37 (0.33)	0.35 (0.31)	0.40 (0.30)
Level of surgical procedure			
1	80/135 (59.3)	82/136 (60.3)	82/134 (61.2)
2	53/135 (39.3)	49/136 (36.0)	46/134 (34.3)
3	2/135 (1.5)	5/136 (3.7)	6/134 (4.5)

Abbrevations: ASA, American Society of Anesthesiologists; BL, bilateral laminotomy; BMI, body mass index (calculated as weight in kilograms divided by height in meters squared); EQ-5D, EuroQol 5-dimensional questionnaire utility index; HSCL: number of patients operated in a different level; HSCL-25, Hopkins Symptom Checklist-25; NRS, Numerical Rating Scale, which ranges from 0 (no pain) to 10 (worst pain imaginable); ODI, Oswestry Disability Index; SPO, spinous process ostetomy; UL, unilateral laminotomy with crossover; ZCQ, Zurich Claudication Questionnaire.

^a^ 3 years or more in college or university.

^b^ ODI ranges from 0 (no impairment) to 100 (the greatest impairment).

^c^ NRS, Numerical Rating Scale ranges from 0 (no pain) to 10 (worst pain imaginable).

**Figure 2.**
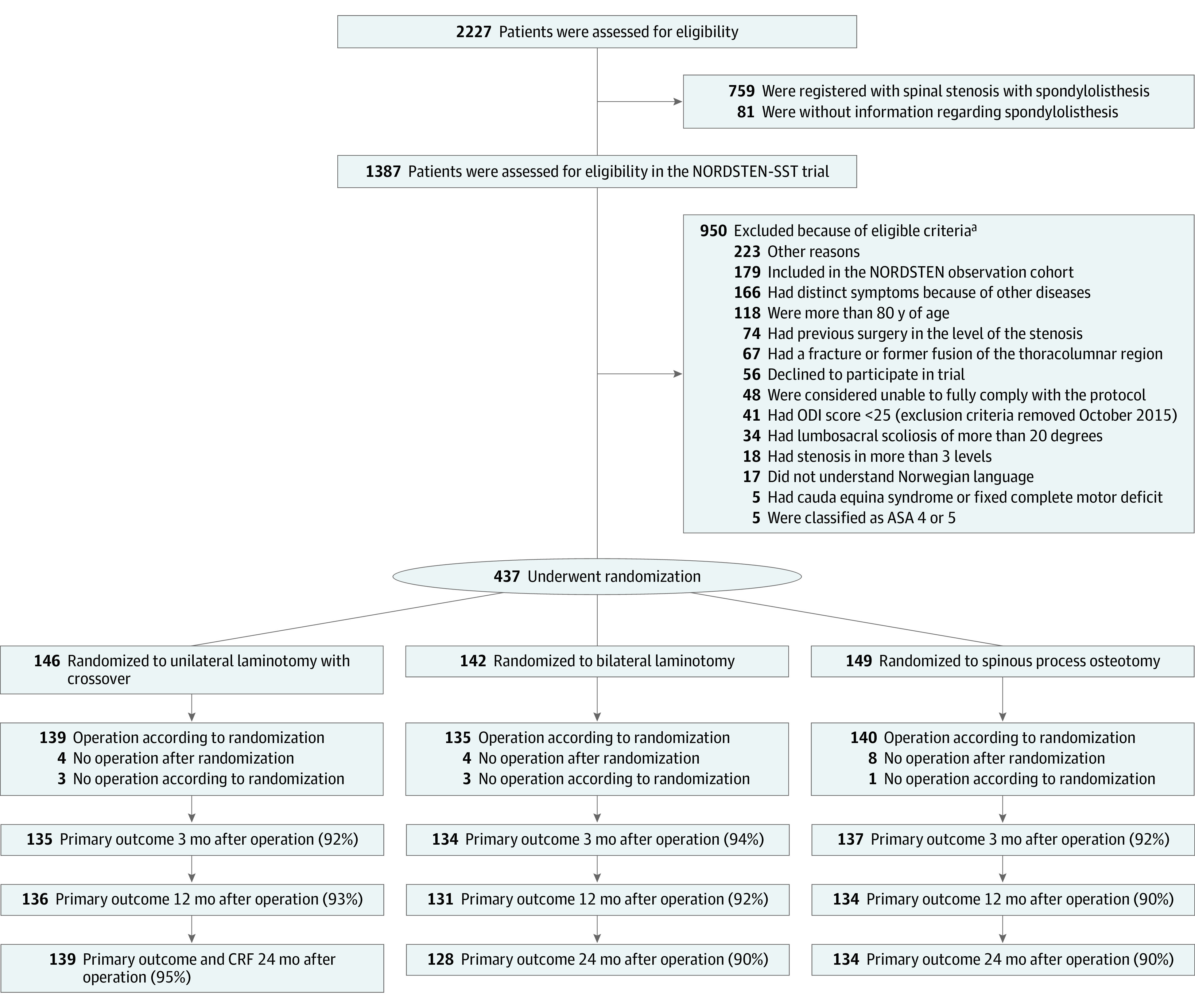
Study Flowchart ASA indicates American Society of Anesthesiologists; CRF, case report form; NORDSTEN, Norwegian Degenerative Spondylolisthesis and Spinal Stenosis study; ODI, Oswestry Disability Index; SST, Spinal Stenosis Trial. ^a^More than 1 exclusion criteria were noted.

Of 2227 patients assessed for eligibility in the NORDSTEN-study, 1387 were eligible for inclusion in the SST, 950 did not fulfill all eligibility criteria and were excluded, and 437 underwent randomization. Figure 2 presents the study.^34^

### Primary Outcome

**Figure 3.**
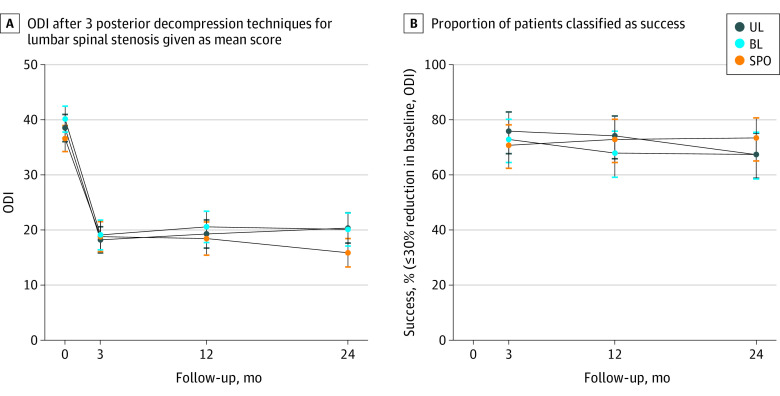
Oswestry Disability Index After 3 Posterior Decompression Techniques for Lumbar Spinal Stenosis Given as Mean Score and Proportion of Patients Classified as Success Patients classified as successs had a reduction in baseline scores 30% or more. BL indicates bilateral laminotomy; SPO, spinous process osteotomy; UL, unilateral laminotomy with crossover.

The actual mean change in ODI from baseline to 2-year follow-up for the whole cohort was −19.1 (95% CI, −20.8 to −17.5). The overall proportion of patients whose outcomes were classified as a success was 273 (69.5%). When adjusting for baseline ODI and operating hospital, the estimated mean change in ODI after 2 years was −17.8 (95% CI, −20.3 to −15.3) for the UL group, −18.7 (95% CI, −21.3 to −16.0) for the BL group and −21.0 (95% CI, −23.5 to −18.4) for the SPO group (Table 2). There were no statistically significant differences between the 3 surgical method groups in the primary outcome (*P* = .214). The mean ODI-score in each of the 3 surgical groups from baseline to 3, 12, and 24 months of follow-up appear in Figure 3 and eTable 2 in Supplement 2. Imputing missing data, the estimated mean (SD) changes in ODI for UL, BL, and SPO were −18.1 (95% CI, −20.7 to −15.6) points, −18.6 (95% CI, −21.1 to −15.9) points, and −21.2 (95% CI, −23.7 to −18.6) points, respectively. Analyzing interaction effects between study arms and period of inclusion, level of the surgical procedure, and number of levels operated did not show any evidence of differential effects of surgical technique in these subgroups of patients (eTable 7 in Supplement 2).

**Table 2.** Primary and Secondary Outcomes^a^

Outcomes	Mean (95% CI)			*P* value	No. eligible for analyses
	Unilateral laminotomy with crossover	Bilateral laminotomy	Spinous process osteotomy		
Primary outcome					
Change in ODI after 24 mo	−17.8 (−20.3 to −15.3)	−18.7 (−21.3 to −16.0)	−21.0 (−23.5 to −18.4)	.21	393
Secondary outcomes					
Proportion success after 24 mo, %	67.4 (53.6 to 81.3)	67.5 (53.1 to 81.8)	73.5 (58.9 to 88.1)	.80	393
Change in global EQ-5D score	0.31 (0.26 to 0.37)	0.31 (0.26 to 0.36)	0.35 (0.30 to 0.40)	.54	358
Change in ZCQ symptom score	−0.96 (−1.10 to −0.83)	−1.02 (−1.16 to −0.88)	−1.09 (−1.23 to −0.96)	.41	389
Change in ZCQ physical function score	−0.79 (−0.89 to −0.69)	−0.85 (−0.95 to −0.74)	−0.91 (−1.01 to −0.80)	.30	390
Change in NRS leg pain score	−3.29 (−3.77 to −2.82)	−3.61 (−4.10 to −3.13)	−3.62 (−4.10 to −3.15)	.55	377
Change in NRS low back pain score	−2.59 (−3.05 to −2.13)	−2.42 (−2.89 to −1.94)	−2.96 (−3.43 to −2.50)	.25	380
Global perceived effect score after 24 mo	2.55 (2.32 to 2.78)	2.55 (2.31 to 2.79)	2.29 (2.06 to 2.52)	.21	398
Duration of procedure, min	95.7 (81.1 to 110.3)	123.9 (109.0 to 138.7)	92.9 (78.2 to 107.7)	<.001	416
Length of hospital stay, d	2.84 (2.18 to 3.50)	3.17 (2.51 to 3.84)	3.09 (2.43 to 3.75)	.38	363
Blood loss, mL	139.0 (96.9 to 181.1)	173.1 (130.8 to 215.4)	150.7 (107.7 to 193.6)	.15	373
Proportion reoperated, %	7.9 (2.2 to 13.7)	4.6 (4.3 to 8.8)	8.2 (2.2 to 14.2)	.44	416
Proportion incidental dural tear, %	5.8 (1.6 to 10.0)	7.5 (2.6 to 12.5)	7.8 (2.9 to 12.6)	.79	402
Proportion wound infection, %	0 (NA)	0.8 (0 to 2.3)	0.0 (NA)	.32	396
Proportion hematoma requiring reoperation, %	1.0 (0 to 3.4)	1.0 (0 to 3.5)	1.9 (0 to 5.8)	.81	397
Proportion other complications, %^b^	1.5 (0 to 3.5)	5.5 (1.4 to 9.5)	4.5 (0.9 to 8.1)	.18	397
Proportion neurological deterioration, %	2.2 (0 to 4.8)	1.6 (0 to 3.8)	0.7 (0 to 2.2)	.59	395

Abbreviations: EQ-5D, EuroQuol 5-dimension questionnaire utility index; NRS, numeric rating score; ODI, Oswestry Disability Index; ZCQ, Zurich Claudication Questionnaire.

^a^ Means and corresponding 95% CI calculated by estimating marginal effects after fitting multilevel linear models with random intercepts for operating hospital and adjusting for baseline measure when analyzing change scores. Proportions and corresponding 95% CI calculated by estimating marginal effects after fitting multilevel Poisson models with random intercepts for operating hospital.

^b^ Other complications include cardiovascular, venous thromboembolism, urological, and respiratory complications.

### Secondary Outcome

There were no statistically significant differences in change score for the secondary outcomes between the 3 surgical groups. Results from analyses on secondary outcomes are given in Table 2 and eFigure 1 and eTable 3 in Supplement 2. In the whole cohort, there was a mean improvement in EQ-5D of 0.32 (95% CI, 0.28-0.36). The mean change in ZCQ was 1.02 (95% CI, 0.94-1.11) for symptom severity and 0.85 (95% CI, 0.78-0.92) for physical function. Likewise, the mean improvement from baseline in NRS was 3.5 (95% CI, 3.2-3.8) for leg pain and 2.7 (95% CI, 2.4-3.0) for back pain.

The BL group had a longer mean duration of the surgical procedure, 123.9 (109.0-138.7) minutes compared with 95.7 (81.1-110.3) minutes and 92.9 (78.2-107.7) minutes for UL and SPO, respectively, (*P* < .001). For other relevant outcomes, there were no differences between the 3 surgical methods. All results related to the surgical procedures are given in Table 2. The mean duration of the surgical procedure was 101 (95% CI, 96-108) minutes for the total cohort, and the length of hospital stay was 3.1 (95% CI, 2.9-3.4) days. The total rate of reoperations in the whole cohort during the 2-year follow up period were 6.4% (95% CI, 4.3%-9.1%), the number of reoperations was 11 of 146 (7.5%) in the UL group, 6 of 142 (4.2%) in the BL group, and 11 of 149 (7.4%) in the SPO group. Overview of the reoperations during the hospital stay, until 3 months follow up, and finally until 2 years of follow up (eTable 4 to eTable 6 in Supplement 2).

## Discussion

The results of this study found no association in favor of any of the 3 most commonly used minimally invasive decompression techniques for LSS in terms of effectiveness. We found no clinically relevant or statistically significant association in mean improvement regarding pain and disability or the proportion of patients reporting clinically important changes for the 3 treatment groups after 2 years (eFigure 2 and eFigure 3 in the Supplement). We also found no association that suggested effectiveness varied by level of the surgical procedure or number of levels operated. The results of the secondary patient-reported outcomes were in line with the primary outcome. We found no significant differences in outcomes related to the surgical procedure, such as length of hospital stay, perioperative blood loss, and perioperative complications. The SPO and UL procedures required approximately 30 minutes less than BL. These findings correspond with those of previous trials with smaller numbers of patients,^11,12,13,14,15,16,17^ both regarding improvement of patient-reported outcome measures and complication rates.

The main strength of the current study is the randomized design and high number of patients (eFigure 4 in the Supplement). Furthermore, the high rate of follow-up improves the internal validity. The external validity would be robust because of the pragmatic inclusion criteria and a large number of highly specialized and smaller orthopedic and neurosurgical centers from all over the country, participating in the inclusion and treatment of the patients. The baseline characteristics and the improvement in disability at 12 months were similar to a previous prospective cohort study from the Norwegian Registry for Spine Surgery,^13^ further indicating a strong external validity. Other strengths are the public financing of the study, blinding of the person who performed the statistical analyses, and the use of an independent study monitor according to ICH-GCP.

There was no statistical difference between the 3 groups regarding the proportion of reoperations during the primary hospital stay, after 3 months, or after 2 years. A 2-year follow-up period is probably insufficient for a complete evaluation or conclusion to be drawn. The study group plans to follow this cohort for 10 years to evaluate this topic more thoroughly. The number of reoperations will also reflect the durability of the various procedures, an important aspect of the effectiveness evaluation.

### Limitations

This study has limitations. The minimally invasive surgical methods evaluated in this study are not compared with a full laminectomy. All the midline retaining procedures could potentially be important to avoid postlaminectomy spondylolisthesis.^9,35,36^ The reason for not including the laminectomy method was that most of the centers already had stopped using this technique and used the 3 minimally invasive techniques as standard methods. Moreover, a study from the Norwegian Registry for Spine Surgery has shown similar results after minimally invasive decompression and full laminectomy.^10^

A decompressive procedure is performed to relieve the dural compression at the affected level of the spine and be comprehensive enough to achieve sufficient symptom relief. A secondary radiological study from this trial reported that UL, BL, and SPO provided a similar increase of the dural sac cross-sectional area (DSCA).^37^ Hence, both radiological and clinical outcomes seem similar in the surgical techniques compared 2 years postoperatively. The outcome for these patients will be followed up with for 10 years to investigate eventual changes over time.

Mannion et al^38^ reported that a high degree of stenosis preoperatively was associated with a better outcome after the surgical procedure. However, it is unclear how extensive the increase of DSCA needs to be to obtain long-term symptom relief. One study has shown an association between a large increase of DSCA postoperatively and patient-reported outcome,^39^ but 2 studies did not confirm these findings.^14,40^ In our opinion, it has not been established whether a wide decompression yields superior clinical results compared with less extensive decompression. The threshold value for the decompression size will be addressed in a future study. In our opinion, it is important to differentiate the effect of the surgical procedure and to evaluate the impact of the decompression method used in terms of the effect on the surrounding structures, including stability of the spine and muscular damage. This will also be addressed in further studies from the NORDSTEN-SST cohort.

The 3 different surgical techniques vary in how much the surrounding tissue is affected. BL requires a bilateral release of the multifidus muscle, and SPO requires an osteotomy of the spinous process. The degree of surgical trauma can affect postoperative fibrosis of the muscles and nerve innervation. The equivalent clinical result from the present study indicates that the surgical impact of the surrounding tissue is of minor importance and that other factors concerning the surgical outcome must be assessed to improve the results after the surgical procedure for LSS.

The change in inclusion criteria, including patients with a baseline ODI of fewer than 25 points, might be seen as a limitation. To investigate the robustness of our findings, we performed analyses studying the effect of surgical technique by timing of study inclusion (before or after November 1, 2015). No evidence suggesting differential effects by inclusion period were found (eTable 7 in Supplement 2).

The actual sample size was somewhat lower than initially planned, which would reduce the statistical power of the study. Originally, we planned our study with a 15% drop-out, corresponding to an actual sample size of 135 participants per study arm. However, the drop-out rate turned out to be lower so the number of participants eligible for analyzing the primary outcome was 393, corresponding to a sample size of 131 per arm. With the same a priori assumptions, this sample size would give an estimated power of 79%, only marginally lower than the required 80%.

## Conclusions

In the present trial of patients treated surgically for lumbar spinal stenosis, there were no differences in the effectiveness between the 3 most commonly used minimally invasive posterior decompression techniques. The complication rates did not differ among the 3 methods, although surgical time differed among them.

## Notes

Supplement 1. **eAppendix 1.** The NORDSTEN-Study Spinal Stenosis Trial-SST Protocol **eAppendix 2.** Norwegian Spinal Stenosis Study Research Protocol

Supplement 2. **eTable 1.** Inclusion and Exclusion Criteria for the Spinal Stenosis Trial (SST) in the NORDSTEN-Study **eTable 2.** Primary Outcome **eTable 3.** Secondary Outcome **eTable 4.** Reoperations During the Hospital Stay **eTable 5.** Reoperation Recorded After Discharge and Until 3 Months of Follow Up **eTable 6.** Reoperation Recorded After 3 Months and Until 24 Months of Follow Up **eTable 7.** Interactions **eFigure 1.** Secondary Outcomes After 3 Posterior Decompression Techniques for Lumbar Spinal Stenosis **eFigure 2.** Global Perceived Effect **eFigure 3.** Distribution of the Primary Outcome (Change in ODI) for Each Study Arm **eFigure 4.** Cumulative Number of Included Patients Over Time **eAppendix 1.** The NORDSTEN-SST Study Monitor Report **eAppendix 2.** The NORDSTEN Collaboration
eReferences.

Supplement 3.
Data Sharing Statement
