# Supplementary material for: Comparison of 3 Different Minimally Invasive Surgical Techniques for Lumbar Spinal Stenosis: A Randomized Clinical Trial
Source: JAMA Netw Open. 2022 Mar 28;5(3):e224291. doi: 10.1001/jamanetworkopen.2022.4291 (PMC8961320; doi:10.1001/jamanetworkopen.2022.4291)
Supplement: Supplement 1. — eAppendix 1. The NORDSTEN-Study Spinal Stenosis Trial-SST Protocol eAppendix 2. Norwegian Spinal Stenosis Study Research Protocol [file jamanetwopen-e224291-s001.pdf]

The NORDSTEN-STUDY Spinal Stenosis Trial-SST Protocol

Statistical Analysis Plan

Comparison of different surgical treatments for Lumbar Spinal Stenosis. A randomized controlled trial comparing the clinical and radiological results using "unilateral laminotomy with crossover", "bilateral laminotomy" and "spinous process osteotomi".

The NORDSTEN-study

Spinal Stenosis Trial-SST.

NCT02007083

Participating authors:

Erland Hermansen, PhD (Corresponding author) (1,2), E-mail: erland.hermansen@helse-bergen.no

Ivar Magne Austevoll PhD, E-mail: imau@helse-bergen.no

Tor Åge Myklebust PhD, (3), E-mail: tor.age.myklebust@helse-mr.no

Frode Rekeland, MD, E-mail: frode.rekeland@helse-bergen.no

Tore Solberg PhD, E-mail: tsdrugiss2@gmail.com

Kjersti Storheim Professor, E-mail: UXSKJE@ous-hf.no

Oliver Grundnes, PhD, E-mail: oliver.grundnes@ahus.no

Jens Ivar Brox Professor, E-mail: j.i.brox@medisin.uio.no

Christian Hellum PhD, E-mail: christian.hellum@ous-hf.no

Kari Indrekvam, PhD, (2) E-mail: kari.indrekvam@helse-bergen.no

(1) Department of Orthopedic Surgery, Ålesund Hospital, Møre and Romsdal Hospital Trust.

(2) Kysthospitalet in Hagevik. Orthopedic Clinic, Haukeland University Hospital, Bergen.

(3) Department of Research and Innovation, Møre and Romsdal Hospital Trust, Ålesund, Norway.

1) Administrative Information

This document is a supplement to the NORDSTEN-SST protocol, "Comparison of different surgical treatments for Lumbar Spinal Stenosis. A randomized controlled trial comparing the clinical and radiological results using "unilateral laminotomy with crossover", "bilateral laminotomy" and "spinous process osteotomi" [1].
The SST is one of three arms in the NORwegian Degenerative spondylolisthesis and spinal STENosis (NORDSTEN)- study. The two other arms are Degenerative Spondylolisthesis Trial (DST) and the Observational Cohort (OC).

Trial Registration Number

The trial is registered in ClinicalTrials.gov, first posted december 10, 2013 (Identifier: NCT02007083).

Sponsor/ Funding

The project has received funding from Helse Vest RHF (the Western Regional Health Authority) and Helse Midt RHF (Central Norway Regional Health Authority). The funder has no influence in study design, management and interpretation of data or in decision to submit data.

Justification for SAP revision:

Update January 2016 From the start of inclusion (April 15, 2014) patients with ODI scores less than 25 were excluded. Due to experiences from participating surgeons that a considerable part of the patients was excluded due to ODI less than 25, even if their complaints from leg and back did justify an operation. To enhance the external validity of the study, the steering committee decided that from date 29th August 2015, the patients should not be excluded due to ODI- score lower than 25.

2) Introduction

Background and rationale

The clinical entity “Lumbar Spinal Stenosis” can be defined as a narrowing of the lumbar spinal canal with its contents. This leads to compression of nervous and vascular structures.

The surgical treatment of LSS is to perform a decompression. Several studies have been conducted to compare different posterior decompression techniques. A Cochrane review article from 2015 stated that one cannot conclude which procedure give superior clinical results in these studies[2], Jacobs et al published a review, which includes four previous review papers (among a previous Cochrane-review), and concluded that the scientific evidence was of low or very low quality, and that there was no evidence to recommend one particular surgical method over another, or the extent of decompression needed [3].

Therefore, we plan to perform a multicentre, randomized trial to compare the clinical and radiological results of three midline retaining posterior decompression techniques;“spinous process osteotomy” (SPO), “bilateral laminotomy” (BL), and “unilateral laminotomy with crossover” (UL).

Objectives

Main Objective

The primary objective is to investigate whether one of the three posterior decompression techniques gives superior results vs the two other surgical methods, and to investigate the extent of decompression needed to sustain good long-term clinical results.

Secondary objectives

Predictor analysis

To evaluate whether selected radiological parameters and patient characteristics masured at baseline can predict outcomes, and thereby also used to choose the most appropriate treatment.

3) Study Methods

Trial design

The NORDSTEN-SST is an open label, multicentre randomized controlled trial, with a superiority design, and three parallel groups. The patients will be randomized into on of three surgical methods;

Group A) Unilateral laminotomy with crossover (UL)

The laminotomy is first performed ipsilaterally. The decompression of the spinal canal is initiated by a flavectomy followed by a laminotomy of the lower part of the superior lamina, and the upper part of the inferior lamina. Laterally, a medial facetectomy is performed and the patient is then slightly rotated to visualize the contralateral side. The dura is retracted, and the decompression is performed contralaterally.

Group B) Bilateral laminotomy (BL)

The decompression of the spinal canal is initiated by a bilateral flavectomy followed by a bilateral laminotomy of the lower part of the superior lamina, and the upper part of the inferior lamina. Laterally, a medial facetectomy is performed,

Group C) Spinous process osteotomy (SPO)

An osteotomy is performed, at the base of the spinous process above (and sometimes under) the affected level. The spinous process is retracted to the contralateral side with intact supraspinal and interspinal ligaments, giving a midline access to the spinal canal. The decompression is first performed in the midline, then laterally at both sides. A laminotomy of the lower part of the superior lamina and the upper part of the inferior lamina is performed, followed by a medial facetectomy. Both nerve roots are visualized, and the lateral recesses are decompressed. Special attention is warranted when a multilevel decompression is performed to retain at least 1/3 of the lamina.

The SPIRIT checklist [4] has been used as a template for the Study protocol [1]. The reporting of the trial will be based on an adapted Consolidated Standards of Reporting Trials (CONSORT) checklist for randomized trials [5].

Randomization

Eligible patients are randomised into one of three arms, unilateral laminotomy with crossover, bilateral laminotomy and spinous process osteotomy with a 1:1:1 allocation. A randomized block design, stratified by hospital and the blocks made as small as possible, is used to ensure equal distribution of all three treatments (randomly selected block size 4 and 6). Randomisation is performed in the 6 weeks before surgery. The randomisation procedure is concealed and administered by a study coordination centre at a university hospital and communicated to a local research coordinator who is not involved in the treatment of the patients. Randomisation is performed after the patient has signed the informed consent form. The result of the randomisation is documented in the patient record. Hence, neither the patients nor the surgeons can influence the type of intervention.

Sample size

The trial is planned with a superiority design. The study is designed to detect a difference of 7 ODI-points between the groups. With a standard deviation of 18, a significance level of 0.02, 80% power and a drop-out rate of 15%, we need to include 155 patients in each group. Thus, we plan to include 465 patients over a 4-5 year period. The analysis will be performed according to the intention to treat principles. If the number of drop-out or missing data exceeds 15%, imputation will be performed

Outcome assessment

In each hospital, non-blinded coordinators (not surgeons) will ensure that the questionnaires are completed at baseline, 3 months, 12 months, 2 years, 5 years and 10 years.

Patient reported outcome measures (PROM)

1) Primary outcome:

The primary outcome is change in the Oswestry Disability Index (ODI) Norwegian version 2.0 [6] from baseline to 2-year follow-up. The ODI includes 10 questions about pain and activities of daily living. Each item has five response categories from no pain related disability (0) to the worst possible pain related disability (5). Responses are transformed into

an index ranging from no disability (0) to the worst possible disability (100). The ODI questionnaire is the most widely used and validated outcome measure in spinal surgery [7] [8].

2) Secondary outcomes:

Secondary patient reported outcomes are changes from baseline to follow-up in the EuroQol 5-dimensional questionnaire utility index (EQ-5D), the Zurich Claudication Questionnaire (ZCQ-score), a ten point Numeric Rating Scale (NRS) for low back pain and for leg-pain, and a global perceived effect scale. Here changes at 2, 5 and 10 years will be analysed.

The EQ-5D is a generic measure of health-related quality of life. Five domains are rated: mobility, self-care, activity, pain and anxiety, each by three response categories to provide a utility index ranging from -0.59 (worst possible) to 1.0 (best possible). In addition EQ-VAS provides a single score of the patient`s health condition. EQ-5D is validated for the Norwegian population. Despite its large measurement error it is often used in research for spinal conditions [9, 10]. The ZCQ is a disease specific questionnaire for lumbar spinal stenosis [11]. It includes symptom severity, physical activity and patient satisfaction during follow-up. The global perceived effect scale is a seven point scale, which is recommended for clinical trials of chronic pain conditions [12]. It has six response categories: 1 = completely recovered, 2 = much improved, 3 = slightly improved, 4 = no change, 5 = slightly worse, 6 = much worse and 7 = worse than ever. All questionnaires are validated for lumbar spinal stenosis patients [10, 11, 13] and are in close accordance with recommended PROMs for the study of low back pain conditions [14].

We will also compare the proportion of patients classified as success, between the groups. Based on change in ODI score after the operation, the patients will be dichotomized into success and non-success groups. A success is defined as a patient with an improvement in ODI score of at least 30%. This value is based on a national register study from the Norwegian Registry for Spine Surgery (NORSpine), and is also in accordance with recommendations from the IMMPACT group, when comparing clinical effect between groups [15].

The local study coordinator will record complications and adverse events, length of hospital stay, duration of surgery, blood loss and the need for blood transfusion.

Radiological evaluations:

Radiological evaluations will be performed by independent investigators.

Preoperative and 3 months postoperative MRI scans will be evaluated to measure the extent of decompression by calculating the change in dural sac cross-sectional area at the most stenotic level (square millimetres [16]. The association between the increase in dural sac cross-sectional area and clinical improvement (primary outcome) will be assessed. We will compare the increase in dural sac cross-sectional area in patients who achieve a minimal clinically important change of the ODI with those who do not.

4)STATISTICAL CONSIDERATIONS

Adherence and protocol deviations

The trial is monitored following the Helsinki Declaration, The International Conference on Harmonisation Guideline for Good Clinical Practice (ICH GCP) [17]. An independent monitor, without influence on the scientific work, will be responsible for the monitoring. Due to the nonregulated ICH GCP guideline for this trial (not including drug intervention) the risk and safety will be safeguarded at the same level as data quality. All informed consent forms will be checked, and all registrations of serious events will be monitored. According to the monitoring plan selected variables will be checked. All hospitals will be visited regularly. Adapted versions of the ‘Investigator’s Site File (ISF)’ and the ‘Trial Master File (TMF)’ will be checked for essential documents during the trial. Queries and deviations will be recorded and reported, and the coordinators at responsible hospitals have two months to send a written report with the required corrections to the monitor. All deviations from the protocol will subsequently be recorded at the ‘Note to file form’. Recorded deviations will be presented in the final manuscript, tables or in Supplementary. Statistical Analysis Plan for NORDSTEN-SST, SAP Version 4.2 The patients have major deviations from protocol if they:

- have not received operative treatment in accordance with randomized allocation.
- have received operative treatment in accordance with randomized allocation and operated with a new operation at same level during the follow-up period.
- have not provided informed consent.
- have withdrew the informed consent and claimed their data withdrawn from analyses.

Statistical analyses

All statistical analyses will be performed by a statistician blinded to the treatment given. Since this is a large randomized controlled trial, we will likely not need to adjust for potential confounding factors. However, if descriptive statistics reveal that there are imbalances in the study groups we will make comparisons with proper adjustments for such imbalances.

When comparing changes in outcomes from baseline to a fixed time point (e.g. 2, 5 or 10 years) we will use ANOVA combined with post-hoc testing for continuous outcomes. For categorical outcomes we will use standard chi-square analyses. If there is a need for confounder adjustment we will estimate the proper regression models dependent on the level of measurement of the dependent variable. Multilevel models might be used to analyse repeated measures data.

When trying to identify factors that could serve as predictors for outcome we will estimate multivariable regression models. The following factors are a priori considered to be the most relevant: treatment group, age, sex, BMI, smoking, preoperative dural sac cross-sectional area (in mm^2), percentage achieved decompression, preoperative ODI-score and preoperative NRS-score for leg pain and back pain. Bayesian information criterion (BIC) might be used as a statistical tool to select the best performing model.

5) Trial population

Patients will be recruited by each of the participating hospitals. If there is an indication for bilateral decompression of the spinal canal, and the patient is referred for surgical treatment to one of the participating hospitals, the patient is eligible for inclusion in the study. Inclusion and exclusion criteria are evaluated, and the patient is invited to participate in the study if all inclusion criteria and no exclusion criteria are present. If the patient agrees to participate, he or she will be randomized into one of the three surgical procedures. The patients should have an MRI-scan, and a x-ray of the lumbosacral spine, taken up to 6 months earlier.

Inclusion criteria:

- have clinical symptoms of spinal stenosis as neurogenic claudication or radiating pain into the bilaterally to the lower limbs,
- -not responding to at least 3 months of non-surgical treatment.
- **-**radiological findings corresponding to the clinical symptoms of LSS. Central-stenosis, or lateral recess-stenosis.
- be able to give informed consent and to answer the questionnaires.
- -over 18 years of age
- be able to understand Norwegian language, spoken and in writing

Exclusion criteria:

- have a degenerative lumbar spondylolisthesis, with a slip ≥ 3 mm verified on standing plain x-rays in lateral view.
- -are not willing to give written consent.
- -have former surgery in the level of stenosis.
- -fracture, or former fusion of the thoracolumbal region.
- -cauda equina syndrom (bowel or bladder dysfunction) or fixed complete motor deficit.
- -are ASA- classified 4 or 5.
- -are older than 80 years
- -have a lumbosacral scoliosis more than 20 degrees verified on AP-view
- -have **distinct** symptoms in one or both of their legs due to other diseases, e.g. polynevropathy, vascular claudication or osteoarthtritis.
- -LSS in 4 or more levels.

- -not able to comply fully with the protocol, including treatment, follow-up or study precedures (psychosocially, mentally and physical).
- the patient is participating in another clinical trial that may interfere with this trial

Timeline:

Before operation:

- Skeletal X- rays.
- MRI- scan for measuring preoperatively DSCSA.
- Following completion of the consent form the following PRO- data will be collected: ODI, ZCQ, EQ- 5D, HSCL 25, VAS for back- and leg-pain demographics, work status, smoking status and use of analgesics.

During the hospital stay:

- The surgeon will register radiological findings, ASA-classification, operation method, operation time, level and blood loss, and per-operative complications.
- Complications will be registered during follow-up by an independent nurse or physician.

3 months postoperatively (± 2 weeks):

- The following PRO- data will be collected: Working status, ODI, ZCQ, EQ-5D, VAS for back- and leg-pain, patient satisfaction with treatment outcome, and patient satisfaction with treatment care.
- MRI scan to measure DSCSA postoperatively.

12 months postoperatively (±1 month):

- -The same PRO-data as at 3 months will be collected.

2 years postoperatively (± 2months):

- -The same PRO- data as at 3 months will be collected.
- MRI scan to measure DSCSA.
- -Skeletal x-rays: standard images will be performed.
- -CT- scanning for examining the union of the spinous process (only the SPO-group).

5 years postoperatively (± 3 months):

- -The same PRO-data as at 3 months will be collected.
- MRI scan to measure DSCSA.
- -Skeletal x-rays: standard images will be performed.

10 years postoperatively (± 3 months):

- -The same PRO-data as at 3 months will be collected.
- Skeletal x-rays: standard images will be performed.

Reference List

1. Hermansen E, Austevoll IM, Romild UK, Rekeland F, Solberg T, Storheim K, Grundnes O, Aaen J, Brox JI, Hellum C *et al*: **Study-protocol for a randomized controlled trial comparing clinical and radiological results after three different posterior decompression techniques for lumbar spinal stenosis: the Spinal Stenosis Trial (SST) (part of the NORDSTEN Study)**. *BMC musculoskeletal disorders* 2017, **18**(1):121.
2. Overdevest GM, Jacobs W, Vleggeert-Lankamp C, Thome C, Gunzburg R, Peul W: **Effectiveness of posterior decompression techniques compared with conventional laminectomy for lumbar stenosis**. *Cochrane Database Syst Rev* 2015, **3**:CD010036.
3. Jacobs WC, Rubinstein SM, Willems PC, Moojen WA, Pellise F, Oner CF, Peul WC, van Tulder MW: **The evidence on surgical interventions for low back disorders, an overview of systematic reviews**. *EurSpine J* 2013, **22**(9):1936-1949.
4. Agha RA, Altman DG, Rosin D: **The SPIRIT 2013 statement--defining standard protocol items for trials**. *Int J Surg* 2015, **13**:288-291.
5. Hopewell S, Clarke M, Moher D, Wager E, Middleton P, Altman DG, Schulz KF: **CONSORT for reporting randomized controlled trials in journal and conference abstracts: explanation and elaboration**. *PLoSMed* 2008, **5**(1):e20.
6. Fairbank JC, Pynsent PB: **The Oswestry Disability Index**. *Spine (Phila Pa 1976)* 2000, **25**(22):2940-2952.

7. Steinberger J, Skovrlj B, Caridi JM, Cho SK: **The top 100 classic papers in lumbar spine surgery**. *Spine* 2015, **40**(10):740-747.
8. Fairbank JC: **Why are there different versions of the Oswestry Disability Index?** *Journal of neurosurgery Spine* 2014, **20**(1):83-86.
9. Hansson T, Hansson E, Malchau H: **Utility of spine surgery: a comparison of common elective orthopaedic surgical procedures**. *Spine (Phila Pa 1976)* 2008, **33**(25):2819-2830.
10. Solberg TK, Olsen JA, Ingebrigtsen T, Hofoss D, Nygaard OP: **Health-related quality of life assessment by the EuroQol-5D can provide cost-utility data in the field of low-back surgery**. *EurSpine J* 2005, **14**(10):1000-1007.
11. Stucki G, Daltroy L, Liang MH, Lipson SJ, Fossel AH, Katz JN: **Measurement properties of a self-administered outcome measure in lumbar spinal stenosis**. *Spine (Phila Pa 1976)* 1996, **21**(7):796-803.
12. Dworkin RH, Turk DC, Farrar JT, Haythornthwaite JA, Jensen MP, Katz NP, Kerns RD, Stucki G, Allen RR, Bellamy N *et al*: **Core outcome measures for chronic pain clinical trials: IMMPACT recommendations**. *Pain* 2005, **113**(1-2):9-19.
13. Spratt KF, Keller TS, Szpalski M, Vandeputte K, Gunzburg R: **A predictive model for outcome after conservative decompression surgery for lumbar spinal stenosis**. *European spine journal : official publication of the European Spine Society, the European Spinal Deformity Society, and the European Section of the Cervical Spine Research Society* 2004, **13**(1):14-21.
14. Clement RC, Welander A, Stowell C, Cha TD, Chen JL, Davies M, Fairbank JC, Foley KT, Gehrchen M, Hagg O *et al*: **A proposed set of metrics for standardized outcome reporting in the management of low back pain**. *Acta orthopaedica* 2015, **86**(5):523-533.
15. Dworkin RH, Turk DC, McDermott MP, Peirce-Sandner S, Burke LB, Cowan P, Farrar JT, Hertz S, Raja SN, Rappaport BA *et al*: **Interpreting the clinical importance of group differences in chronic pain clinical trials: IMMPACT recommendations**. *Pain* 2009, **146**(3):238-244.
16. Steurer J, Roner S, Gnannt R, Hodler J: **Quantitative radiologic criteria for the diagnosis of lumbar spinal stenosis: a systematic literature review**. *BMCMusculoskeletDisord* 2011, **12**:175.
17. GUIDELINE IHT: **INTERNATIONAL CONFERENCE ON HARMONISATION OF TECHNICAL REQUIREMENTS FOR REGISTRATION OF PHARMACEUTICALS FOR HUMAN USE**.

Norwegian Spinal Stenosis Study Research Protocol

Comparison of different surgical treatments for Lumbar Spinal Stenosis. A randomized controlled trial comparing the clinical and radiological results using "spinous process osteotomi", "bilateral laminotomy" and "unilateral laminotomy with crossover".

This is planned to be a multicenter study with 15 participating Norwegian spine centers. The departments of orthopedics at Oslo University hospital and Kysthospitalet i Hagevik, Haukeland University hospital, will be the principal investigators. Two MDs are connected to the study as doctoral students.

Background, introduction and hypothesis

Surgery for Lumbar Spinal Stenosis (LSS) is the most frequently performed procedure today on the adult lumbar spine [4]. The clinical entity "Lumbar Spinal Stenosis" can be defined as a narrowing of the lumbar spinal canal with its contents. This leads to compression of nervous and vascular structures. LSS is a degenerative condition where the patho-anatomic change involves a bulging disc, hypertrophy of the ligamenti flavi and hypertrophy of the facet-joints. The symptoms are most commonly neurogenic claudication, or sciatic pain, and Low Back Pain. The most stenotic part is at the disc-level. Several studies with long-term follow up have been performed to evaluate surgical treatment versus non-surgical treatment [1,20,36], and they conclude that surgical intervention gives superior results. The surgical procedure performed in these studies is a laminectomy. A Cochrane review article also concludes that surgery is superior to non-surgical treatment [12].

The surgical treatment of LSS is to perform a decompression. To achieve relief of the stenosis, one must remove the flaval ligaments, and the lamina (whole, or parts of it) at the disc level, and carry out a partial medial facetectomy to decompress the nerve-root in the lateral recess. The traditional operation is a laminectomy, which involves the total removal of the lamina and the flaval ligaments, with a medial facetectomy. This procedure also removes much of the posterior ligamenture of the spine, and is considered to be a relatively invasive procedure; it is also prone to postoperative instability of the operated spinal segment. In recent decades bilateral laminotomy has become an alternative to a laminectomy [2,19]. This is a less invasive procedure, in which parts of the lamina are retained, and the interspinal and supraspinal ligaments are mostly kept intact. This procedure is therefore considered to be better at preserving stability. More recently a unilateral decompression has been described [21], referred to as a "bilateral laminotomy with crossover technique". The access to the spinal canal is unilateral, and the ipsilateral side is decompressed as usual, whereas the contralateral side is decompressed using a crossover technique. This has theoretical advantages by preservating more of the paraspinal muscles, ligaments and bony structures. A third alternative is to do a spinous process osteotomy [13]. This procedure also involves unilateral release of the paraspinal mucles, and an osteotomy of the spinous process at the

base, above the actual level. These osteotomies facilitate access to the spinal canal with improved visibility, which may be an advantage over the other methods.

Several studies have been conducted to compare laminectomy and bilateral laminotomies [3,8,11,24,26,34,35]. A Cochrane review article from 2005 state that one cannot conclude which procedure gives superior clinical results in these studies [12]. In our opinion later studies have not fulfilled this purpose either. Some studies compare “bilateral laminotomy” with “unilateral laminotomy with crossover”. One of these studies shows inferior results with “unilateral laminotomy with crossover” [35], and two studies show equal clinical results between the two procedures [6,17]. There are no studies comparing spinous process osteotomy with other procedures.

Therefore, we plan to perform a multicentre, randomized trial to compare the clinical and radiological results of “spinous process osteotomy” (SPO), “bilateral laminotomy” (BL), and “unilateral laminotomy with crossover” (UL).

Clinical results

The main goal of this study is to identify which method gives the best long-term clinical results, including the most pain reduction and the best functional results. The patient’s own, self-evaluated assessment of the surgical procedure is the most important outcome. Today, there are several questionnaires that are used to map the clinical status of the patient. The patient answers a questionnaire before the surgical procedure, and the same questionnaire is repeated after surgery, to monitor the effect of the surgical treatment. We have decided to use the same questionnaires that the Norwegian Quality Register for Spinal Surgery (NORSPine) uses, and thus cooperate with the register when collecting data postoperatively. These questionnaires consists of the Oswestry Disability Index (ODI), EQ-5D, Numeric Rating Scale (NRS) for low back pain (LBP), Numeric Rating Scale (NRS) for leg-pain, and the patient’s self-evaluated effect of surgery. These questionnaires have been evaluated in several studies [7,9,10,15,31]. They give an evaluation of the patient`s pain situation, level of function, perceived quality of life, and the self-assessed effect of the surgical treatment. In addition we will use the Swiss Spinal Stenosis Questionnaire (SSSQ). This questionnaire is considered more specific to spinal stenosis, and has also been evaluated in several studies [25,33].

In recent decades the ODI has had a special status within spinal care. There are hardly any studies where clinical results are monitored without this questionnaire. In this questionnaire ‘0’ is the best status, and ‘100’ is the worst status. The desired effect of surgical treatment is a significant decrease in the patient’s ODI-score. We have therefore chosen the ODI as a primary outcome of this study. This application aims to reveal the 10 year improvement in ODI related to the three different surgical methods. When using the ODI as a primary outcome, it is important to decide beforehand what is to be regarded as a significant improvement in the score following surgical treatment. This is now discussed as the Minimal Clinically Important Difference (MCID). MCID is measured as the difference in the mean ODI-scores of patients that score “some improvement” compared with those who score “no improvement”. This value has been found to be 10-12 ODI-points [5,14,23].

Radiological results

Studies have been conducted to identify objective radiological criteria for LSS [32]. We have chosen to investigate the Dural Sac Cross Sectional Area (DSCSA). This is measured at the disc level where the narrowest stenosis occurs. DSCSA can be measured both on a CT-scan and MRI-scan [22,28-30]. The normal population is reported to have a DSCSA of about 170 mm². In the studies quoted, moderate stenosis was defined as a DSCSA between 70 and 100 mm², and significant stenosis as an area below 70 mm². One study found a significant correlation between DSCSA and clinical symptoms, but one other study did not confirm this. Both of these studies included a relatively small number of patients (n=109) [29]. Our previous study [16], has shown a positive correlation between an increase in the DSCSA and improvement in clinical symptoms. To our knowledge this has not been documented before. At present, we cannot identify a threshold value in the spinal canal area that gives clinical symptoms. Neither can we identify a minimal increase in area that is needed to give long-term relief of symptoms. We hope to be able to answer these questions. Our previous study showed an increase in DSCSA using SPO of 101%, up to an average area of 161 mm² postoperatively [16]. Dalgic et al compared “bilateral laminotomy” and unilateral laminotomy with crossover”, and found a postoperative area of 126 mm². This is significantly lower than the area found when SPO is used. We are therefore planning to measure DSCSA before and after surgery, to see whether there are differences between the three different surgical methods. We also want to investigate whether there is a correlation between achieved increase in area and the patients’ self-evaluated outcome of surgery.

Operative differences

As mentioned earlier, there are differences between the three surgical methods. We are planning to observe whether there are significant differences between the methods as regards operation time, amount of peri-operative bleeding, peri-operative complications and length of hospital stay. And, we will also examine whether one of the three surgical procedures have an increased rate of reoperations in the observation period. Thereby we are able to monitor costs of each of the three methods.

We also want to find out whether any of the surgical methods preserves stability better than the others. Preoperative standing x-rays, with lateral view of flexion-extension of the lumbosacral spine are therefore important as a baseline. In the follow up period we will repeat these x-ray investigations and thus find out if any of the methods is more prone to postoperative instability.

Project participants

Project Manager

Clinical director/ Chief surgeon/Assistant Professor Kari Indrekvam.

Indrekvam is project manager and is involved in the planning and implementation of the project, and will be a co-author of any publications. She works at Kysthospitalet in Hagevik,

University Hospital of Bergen, and as an assistant professor at University of Bergen, Department of Surgical Sciences.

Assistant Project Manager

Consultant orthopaedic surgeon, Christian Hellum. He works at Ullevål Hospital, Oslo University Hospital as a consultant surgeon. Hellum is involved in the preparation of the study, and will be a co-author of any publications. He is responsible for the Norwegian Disc-prosthesis study, and is the corresponding author of these publications.

Project administrator/PhD-scolarship

Consultant orthopaedic surgeon Erland Hermansen, is working in the Orthopaedic department, Ålesund Hospital. Hermansen is the corresponding author in our two previous retrospective studies about SPO. He is granted a PhD scholarship for this study.

Project participant: Consultant orthopaedic surgeon Ivar Magne Austevoll.

Project participant: Kjersti Storheim, Dr. scient: Assistant Professor at Oslo University Hospital.

We are planning a multicentre study. The following hospitals will participate:

Oslo University Hospital, Ullevål (OUS), Akershus University Hopsital (AHUS), Stavanger University Hospital (SUS), Kysthospitalet I Hagevik (KiH), University Hospital of North Norway (UNN), Ålesund Hospital. Levanger Hospital, Skien Hospital, Lillehammer Hospital, Gjøvik Hospital, Drammen Hospital, Martina Hansens Hospital, Bærum Hospital, Haukeland University Hospital and Arendal Hospital.

This is a total of 15 hospitals in Norway. A majority of the spinal surgeons in Norway will attend the study in one way or another. Thereby, a unique academic environment will be created.

Steering commite:

We have included persons with a special competence in spinal surgery and spinal research in Norway. Several of the members have a significant amount of publications.

Kari Indrekvam (KiH), Christian Hellum (OUS), Jens Ivar Brox (OUS), Tore Solberg (UNN), Kjertsi Storheim (FORMI), Erland Hermansen (Ålesund Sykehus), Ivar M Austevoll (KiH) and (Mohamed Ahmed (KiH).

Aim of the study

We are planning a multicentre, randomized controlled trial to compare the clinical and radiological results of three different surgical methods for surgical treatment of LSS.

The main objective of the study is to compare the clinical results:

Primary outcome:

The primary outcome is a measurement of the decrease of ODI (version 2.0) in the three different surgical groups. This will be done by calculating the mean ODI improvement in each group after two years, compared to the two other groups.

As a supplement to the primary outcome we will also compare the proportion of responders between the SPO group, the BL group and the UL group, 24 months after the operation. Based on the size of change in ODI score from before to after the operation, we will dichotomize the patients, in a responder group and in a non- responder group. The threshold value for being categorized as a responder will be computed in a planned study based on data from The Norwegian Registry for Spine Surgery (NORSpine).

We are planning to follow the patients for 10 years.

Secondary outcomes:

The secondary outcomes are measurements of change in EQ-5D, SSS-score, NRS for LBP, NRS for leg-pain, and self-evaluated effect of surgery in the three different surgical groups. This will be done by calculating the average values after two years, in each group, and comparing them to the two other groups.

Radiological evaluations:

There are several radiological questions we want to investigate.

-Does one of the methods give a larger increase in DSCSA?

-Is there a correlation between an increase in DSCSA and clinical improvement?

-Does one of the surgical methods give higher incidence of postoperative instability?

Test subjects

Patients will be recruited in each of the participating hospitals. If there is an indication for bilateral decompression of the spinal canal, and the patient is referred for surgical treatment to one of the participating hospitals, the patient is eligible for inclusion in the study. Inclusion and exclusion criteria are evaluated, and the patient is invited to participate in the study. If the patient agrees to participate, he or she will be randomized into one of the three surgical procedures. One of the project participants will attend the surgical procedure, either as the main surgeon or the assistant surgeon. The patients should have an MRI-scan, and a x-ray of the lumbosacral spine, taken up to 6 months earlier.

Inclusion criteria:

- have clinical symptoms of spinal stenosis as neurogenic claudication or radiating pain into the bilaterally to the lower limbs, not responding to at least 3 months of conservative treatment.
-Radiological findings corresponding to the clinical symptoms of LSS. Central -stenosis, or lateral recess-stenosis.
- be able to give informed consent and to answer the questionnaires.
-over 18 years of age

Exclusion criteria:

- have a degenerative lumbar spondylolisthesis, with a slip verified on standing plain x-rays in lateral view.
-are not willing to give written consent.
-have former surgery in the level of stenosis.
-fracture, or former fusion of the thoracolumbal region.-cauda equina syndrom (bowel or bladder dysfunction) or fixed complete motor deficit.
-are addicted to any drugs
-are not able to follow the study protocol
-are classified ASA- > 3.
-are older than 80 years
-have a lumbosacral scoliosis more than 20 degrees verified on AP-view
-have distinct symptoms in one or both of their legs due to other diseases, e.g. polynevropathy, vascular claudication or osteoarthtritis.
-LSS in 4 or more levels.
-not able to comply fully with the protocol, including treatment, follow-up or study precedures (psychosocially, mentally and physical).

We have prepared a separate patient information protocol, which is not presented here.

Research methods and analysis:

We have prepared a separate operation protocol, which is not presented here.

Statistics:

We have previously discussed that MCID is around 10-12 ODI-points for this group of patients. This value indicates the individual effect of treatment versus no-treatment. There is today no consensus in the literature of a clinically important difference between treatment groups. We have therefore decided that we want to reveal a difference of 7 ODI-points between the groups. A statistician has calculated a difference of 7 ODI-points between the treatment groups, and in a regular superiority design. Standard deviation is set to 18. Because we have three groups we want to compare the significans-level is lowered from 5% to 2% (p = 2). The analyzes in the treatment group is done in the following way: -A compares with B, -A compares with C, -B compares with C.). With 80% power , significans level of 2%, and dropout of 30% n in each group is estimated to 187 (131 + 56). 30% dropout is a high

number, but we are planning to monitor the patients for 10 years, so we must anticipate a high dropout number. If we have problems to achieve 187 in each group, we will settle with 15% dropout. This gives 155 patients in each group**.**
We will also perform descriptive analyzes. The superiority analyzes is conducted in a One-way Anova system and Post Hoc.
Therefore we are planning to include 561 patients over a 2-3 year period. The analysis is conducted after the Intention To Treat Principle (ITT). Crossovers will be analyzed in the group they were originally randomized into. A sensitivity analysis will be conducted where patient crossing over from one treatment to another will receive the last score before crossover. Dropouts will be registered with the last value before dropout (last value carried forward).
Department of Clinical Research Support, at Oslo University Hospital will provide the block randomisation, and is performed within a computer program called Medinsight. The randomization process cannot be influenced by participating hospitals, and will be performed within 1 month before treatment.
Patient-data, and registration will also be handled by the computer programme called Medinsight.
The research protocol will be registered in www.clinicaltrials.gov.
The reporting of the trial will be bases on an adapted CONSORT (Consolidated Standards of Reporting Trials) checklist for reporting in randomised trials [18].

Supplementary analysis I

In addition to the main analysis we will investigate whether following predictors will be associated with the success-rate after 24 months: Treatment group, patient age, gender, level of stenosis, segmental instability verified on flexion/extension x-rays,orientaion of the facet joint in the level of stenosis, comorbidity (ASA group),Body Mass Index, preoperative ODI score, smoking and hospital. This analysis will be done by logistic regression.

Supplementary analysis II

All patients that are assessed for eligibility will be registered, and accounted for. This is explained in the CONSORT statement for clinical trials. There will be a group of patients that meet all the inclusion criteria, and have no of the listed exclusion criteria's. However, this group of patients may not have sufficient symptoms to justify surgical treatment. This group of patients will be registered in an observation cohort. We will collect base-line questionnaires of these patients, and perform radiological and clinical assessment. We plan to follow this observational cohort over 10 years. Thereby we will be able to perform a predictor analysis of the patients that experiences deterioration of the clinical condition. They will receive and sign an informed consent form.

Supplementary analysis III

We have previously conducted a study that showed 44% union-rate of the SPO (accepted for publication in Asian Spine Journal). We are planning to repeat this investigation, and evaluate whether the osteotomy of the spinous process has negative implications for the clinical results. A CT scan will therefore be performed two years postoperatively to evaluate the union rate at the operated level.

Ethical considerations:

The patients will receive thorough information both orally and in writing, so that they can give informed consent to the study. All three surgical procedures are well established treatments in Norway today. There is no currently available evidence to suggest that one method is significantly better than the others. In our opinion there are no ethical problems in relation to this study.
We will follow the Helsinki-declaration, ICH Guideline for Good Clinical Practice (ICH GCP).
We have recived an approvel from the Norwegian Commitees for Medical and Health Research Ethics (2011/2034).

Timeline:

Before operation:

-Skeletal X- rays.
-MRI- scan for measuring preoperatively DSCSA.
-Following completion of the consent form the following PRO- data will be collected: ODI, ZCQ, EQ- 5D, VAS for back- and leg-pain demographics, work status, smoking status and use of analgesics.

During the hospital stay:

-The surgeon will register radiological findings, ASA-classification, operation method, operation time, level and blood loss, and per-operative complications.
-Complications will be registered during follow-up by an independent nurse or physician.

3 months postoperatively (± 2 weeks):
-The following PRO- data will be collected: Working status, ODI, ZCQ, EQ-5D, VAS for back- and leg-pain, patient satisfaction with treatment outcome, and patient satisfaction with treatment care.
- MRI scan to measure DSCSA postoperatively.

12 months postoperatively (±1 month):

-The same PRO-data as at 3 months will be collected.

2 years postoperatively (± 2months):

-The same PRO- data as at 3 months will be collected.
Skeletal x-rays: standard images will be performed.

CT- scanning for examining the union of the spinous process (only the SPO-group).

5 years postoperatively (± 3 months):

-The same PRO-data as at 3 months will be collected.
-Skeletal x-rays: standard images will be performed.

10 years postoperatively (± 3 months):

-The same PRO-data as at 3 months will be collected.
-Skeletal x-rays: standard images will be performed.

Flow-chart for Norwegian spinal stenosis study

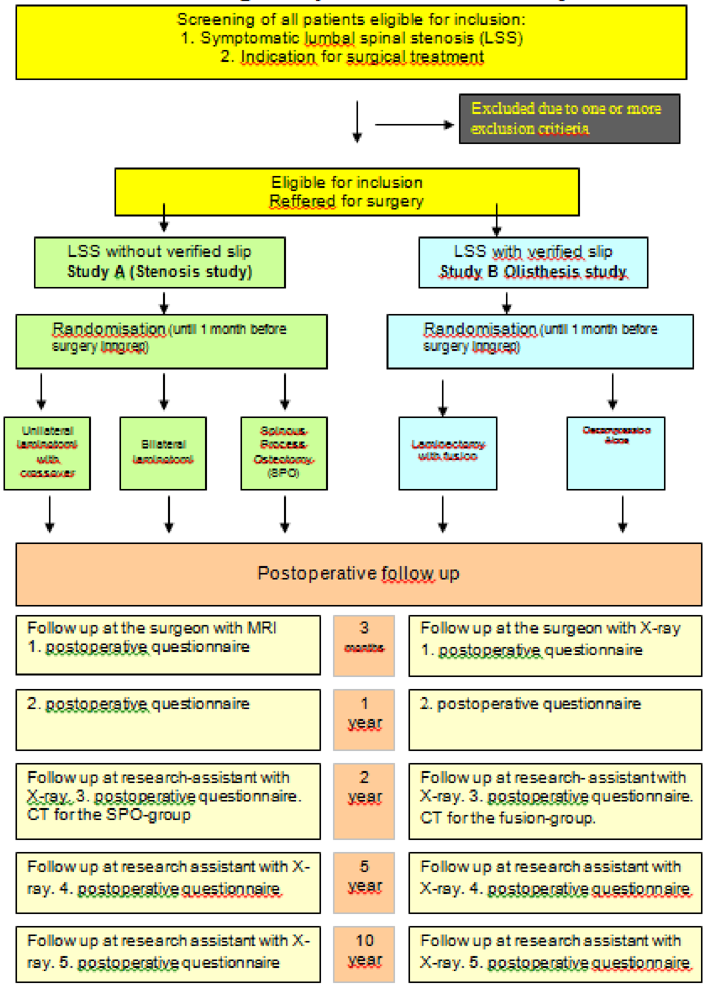

Project budget:

Budget information is described in a separate document.

Monitoring of the trial

Department for Clinical Research Support, at Oslo University Hospital will be monitoring the trial.

References

[1] T Amundsen, H. Weber, H.J. Nordal, B. Magnaes, M. Abdelnoor, F. Lilleas, Lumbar spinal stenosis: conservative or surgical management?: A prospective 10-year study, Spine (Phila Pa 1976.) 25 (2000) pp. 1424-1435.

[2] J Aryanpur and T. Ducker, Multilevel lumbar laminotomies: an alternative to laminectomy in the treatment of lumbar stenosis, Neurosurgery 26 (1990) pp. 429-432.

[3] S E Celik, S. Celik, K. Goksu, A. Kara, I. Ince, Microdecompressive laminatomy with a 5-year follow-up period for severe lumbar spinal stenosis, J. Spinal Disord. Tech. 23 (2010) pp. 229-235.

[4] M A Ciol, R.A. Deyo, E. Howell, S. Kreif, An assessment of surgery for spinal stenosis: time trends, geographic variations, complications, and reoperations, J. Am. Geriatr. Soc. 44 (1996) pp. 285-290.

[5] A G Copay, S.D. Glassman, B.R. Subach, S. Berven, T.C. Schuler, L.Y. Carreon, Minimum clinically important difference in lumbar spine surgery patients: a choice of methods using the Oswestry Disability Index, Medical Outcomes Study questionnaire Short Form 36, and pain scales, Spine J. 8 (2008) pp. 968-974.

[6] A Dalgic, O. Uckun, M.F. Ergungor, O. Okay, E. Daglioglu, G. Hatipoglu, L. Pasaoglu, Y.S. Caglar, Comparison of unilateral hemilaminotomy and bilateral hemilaminotomy according to dural sac area in lumbar spinal stenosis, Minim. Invasive. Neurosurg. 53 (2010) pp. 60-64.

[7] M Davidson and J.L. Keating, A comparison of five low back disability questionnaires: reliability and responsiveness, Phys. Ther. 82 (2002) pp. 8-24.

[8] K S Delank, P. Eysel, J. Zollner, P. Drees, B. Nafe, J.D. Rompe, [Undercutting decompression versus laminectomy. Clinical and radiological results of a prospective controlled trial], Orthopade 31 (2002) pp. 1048-1056.

[9] J C Fairbank and P.B. Pynsent, The Oswestry Disability Index, Spine (Phila Pa 1976.) 25 (2000) pp. 2940-2952.

[10] M A Ferreira-Valente, J.L. Pais-Ribeiro, M.P. Jensen, Validity of four pain intensity rating scales, Pain (2011).

[11] Y S Fu, B.F. Zeng, J.G. Xu, Long-term outcomes of two different decompressive techniques for lumbar spinal stenosis, Spine (Phila Pa 1976.) 33 (2008) pp. 514-518.

[12] J N Gibson and G. Waddell, Surgery for degenerative lumbar spondylosis: updated Cochrane Review, Spine (Phila Pa 1976.) 30 (2005) pp. 2312-2320.

[13] R Gunzburg and M. Szpalski, The conservative surgical treatment of lumbar spinal stenosis in the elderly, Eur. Spine J. 12 Suppl 2 (2003) p.S176-S180.

[14] O Hagg, P. Fritzell, A. Nordwall, The clinical importance of changes in outcome scores after treatment for chronic low back pain, Eur. Spine J. 12 (2003) pp. 12-20.

[15] T Hansson, E. Hansson, H. Malchau, Utility of spine surgery: a comparison of common elective orthopaedic surgical procedures, Spine (Phila Pa 1976.) 33 (2008) pp. 2819-2830.

[16] E Hermansen, G. Moen, J. Barstad, R. Birketvedt, K. Indrekvam, Laminarthrectomy as a surgical approach for decompressing the spinal canal: assessment of preoperative versus postoperative dural sac cross-sectional areal (DSCSA), Eur. Spine J. (2013).

[17] S W Hong, K.Y. Choi, Y. Ahn, O.K. Baek, J.C. Wang, S.H. Lee, H.Y. Lee, A comparison of unilateral and bilateral laminotomies for decompression of L4-L5 spinal stenosis, Spine (Phila Pa 1976.) 36 (2011) p.E172-E178.

[18] S Hopewell, M. Clarke, D. Moher, E. Wager, P. Middleton, D.G. Altman, K.F. Schulz, CONSORT for reporting randomized controlled trials in journal and conference abstracts: explanation and elaboration, PLoS. Med. 5 (2008) p.e20.

[19] T J Kleeman, A.C. Hiscoe, E.E. Berg, Patient outcomes after minimally destabilizing lumbar stenosis decompression: the "Port-Hole" technique, Spine (Phila Pa 1976.) 25 (2000) pp. 865-870.

[20] A Malmivaara, P. Slatis, M. Heliovaara, P. Sainio, H. Kinnunen, J. Kankare, N. in-Hirvonen, S. Seitsalo, A. Herno, P. Kortekangas, T. Niinimaki, H. Ronty, K. Tallroth, V. Turunen, P. Knekt, T. Harkanen, H. Hurri, Surgical or nonoperative treatment for lumbar spinal stenosis? A randomized controlled trial, Spine (Phila Pa 1976.) 32 (2007) pp. 1-8.

[21] M F Oertel, Y.M. Ryang, M.C. Korinth, J.M. Gilsbach, V. Rohde, Long-term results of microsurgical treatment of lumbar spinal stenosis by unilateral laminotomy for bilateral decompression, Neurosurgery 59 (2006) pp. 1264-1269.

[22] O Ogikubo, L. Forsberg, T. Hansson, The relationship between the cross-sectional area of the cauda equina and the preoperative symptoms in central lumbar spinal stenosis, Spine (Phila Pa 1976.) 32 (2007) pp. 1423-1428.

[23] S L Parker, O. Adogwa, A.R. Paul, W.N. Anderson, O. Aaronson, J.S. Cheng, M.J. McGirt, Utility of minimum clinically important difference in assessing pain, disability, and health state after transforaminal lumbar interbody fusion for degenerative lumbar spondylolisthesis, J. Neurosurg. Spine 14 (2011) pp. 598-604.

[24] F Postacchini, G. Cinotti, D. Perugia, S. Gumina, The surgical treatment of central lumbar stenosis. Multiple laminotomy compared with total laminectomy, J. Bone Joint Surg. Br. 75 (1993) pp. 386-392.

[25] R K Pratt, J.C. Fairbank, A. Virr, The reliability of the Shuttle Walking Test, the Swiss Spinal Stenosis Questionnaire, the Oxford Spinal Stenosis Score, and the Oswestry Disability Index in the assessment of patients with lumbar spinal stenosis, Spine (Phila Pa 1976.) 27 (2002) pp. 84-91.

[26] J D Rompe, P. Eysel, J. Zollner, B. Nafe, J. Heine, Degenerative lumbar spinal stenosis. Long-term results after undercutting decompression compared with decompressive laminectomy alone or with instrumented fusion, Neurosurg. Rev. 22 (1999) pp. 102-106.

[27] Rosenbaum, PR Rubin, DB, The Central Role of the Propensity Score in Observational Studies for Causal Effects, Biometrika, 1983, pp. 41-55.

[28] N S Schonstrom, N.F. Bolender, D.M. Spengler, The pathomorphology of spinal stenosis as seen on CT scans of the lumbar spine, Spine (Phila Pa 1976.) 10 (1985) pp. 806-811.

[29] F G Sigmundsson, X.P. Kang, B. Jonsson, B. Stromqvist, Correlation between disability and MRI findings in lumbar spinal stenosis: a prospective study of 109 patients operated on by decompression, Acta Orthop. 82 (2011) pp. 204-210.

[30] M Sirvanci, M. Bhatia, K.A. Ganiyusufoglu, C. Duran, M. Tezer, C. Ozturk, M. Aydogan, A. Hamzaoglu, Degenerative lumbar spinal stenosis: correlation with Oswestry Disability Index and MR imaging, Eur. Spine J. 17 (2008) pp. 679-685.

[31] T K Solberg, J.A. Olsen, T. Ingebrigtsen, D. Hofoss, O.P. Nygaard, Health-related quality of life assessment by the EuroQol-5D can provide cost-utility data in the field of low-back surgery, Eur. Spine J. 14 (2005) pp. 1000-1007.

[32] J Steurer, S. Roner, R. Gnannt, J. Hodler, Quantitative radiologic criteria for the diagnosis of lumbar spinal stenosis: a systematic literature review, BMC. Musculoskelet. Disord. 12 (2011) p.175.

[33] G Stucki, L. Daltroy, M.H. Liang, S.J. Lipson, A.H. Fossel, J.N. Katz, Measurement properties of a self-administered outcome measure in lumbar spinal stenosis, Spine (Phila Pa 1976.) 21 (1996) pp. 796-803.

[34] N W Thomas, G.L. Rea, B.K. Pikul, L.J. Mervis, R. Irsik, J.M. McGregor, Quantitative outcome and radiographic comparisons between laminectomy and laminotomy in the treatment of acquired lumbar stenosis, Neurosurgery 41 (1997) pp. 567-574.

[35] C Thome, D. Zevgaridis, O. Leheta, H. Bazner, C. Pockler-Schoniger, J. Wohrle, P. Schmiedek, Outcome after less-invasive decompression of lumbar spinal stenosis: a randomized comparison of unilateral laminotomy, bilateral laminotomy, and laminectomy, J. Neurosurg. Spine 3 (2005) pp. 129-141.

[36] J N Weinstein, T.D. Tosteson, J.D. Lurie, A.N. Tosteson, E. Blood, B. Hanscom, H. Herkowitz, F. Cammisa, T. Albert, S.D. Boden, A. Hilibrand, H. Goldberg, S. Berven, H. An, Surgical versus nonsurgical therapy for lumbar spinal stenosis, N. Engl. J. Med. 358 (2008) pp. 794-810.
