# Supplementary material for: Comparison of 3 Different Minimally Invasive Surgical Techniques for Lumbar Spinal Stenosis: A Randomized Clinical Trial
Source: JAMA Netw Open. 2022 Mar 28;5(3):e224291. doi: 10.1001/jamanetworkopen.2022.4291 (PMC8961320; doi:10.1001/jamanetworkopen.2022.4291)
Supplement: Supplement 2. — eTable 1. Inclusion and Exclusion Criteria for the Spinal Stenosis Trial (SST) in the NORDSTEN-Study eTable 2. Primary Outcome eTable 3. Secondary Outcome eTable 4. Reoperations During the Hospital Stay eTable 5. Reoperation Recorded After Discharge and Until 3 Months of Follow Up eTable 6. Reoperation Recorded After 3 Months and Until 24 Months of Follow Up eTable 7. Interactions eFigure 1. Secondary Outcomes After 3 Posterior Decompression Techniques for Lumbar Spinal Stenosis eFigure 2. Global Perceived Effect eFigure 3. Distribution of the Primary Outcome (Change in ODI) for Each Study Arm eFigure 4. Cumulative Number of Included Patients Over Time eAppendix 1. The NORDSTEN-SST Study Monitor Report eAppendix 2. The NORDSTEN Collaboration eReferences. [file jamanetwopen-e224291-s002.pdf]

Supplemental Online Content

Hermansen E, Austevoll IM, Hellum C, et al. Comparison of 3 different minimally invasive surgical techniques for lumbar spinal stenosis: a randomized clinical trial. *JAMA Netw Open.* 2022;5(3):e224291. doi:10.1001/jamanetworkopen.2022.4291

eTable 1. Inclusion and Exclusion Criteria for the Spinal Stenosis Trial (SST) in the NORDSTEN-Study
eTable 2. Primary Outcome
eTable 3. Secondary Outcome
eTable 4. Reoperations During the Hospital Stay
eTable 5. Reoperation Recorded After Discharge and Until 3 Months of Follow Up
eTable 6. Reoperation Recorded After 3 Months and Until 24 Months of Follow Up
eTable 7. Interactions
eFigure 1. Secondary Outcomes After 3 Posterior Decompression Techniques for Lumbar Spinal Stenosis
eFigure 2. Global Perceived Effect
eFigure 3. Distribution of the Primary Outcome (Change in ODI) for Each Study Arm
eFigure 4. Cumulative Number of Included Patients Over Time
eAppendix 1. The NORDSTEN-SST Study Study Monitor Report
eAppendix 2. The NORDSTEN Collaboration
eReferences.

This supplemental material has been provided by the authors to give readers additional information about their work.

© 2022 Hermansen E et al. *JAMA Network Open.*

eTable 1. Inclusion and Exclusion Criteria for the Spinal Stenosis Trial (SST) in the NORDSTEN-Study

**Inclusion criteria:**
Clinical symptoms of lumbar spinal stenosis: neurogenic claudication or bilateral radiating pain
Non-responding to at least 3 months of non-surgical treatment
Radiological findings corresponding to the clinical symptoms of LSS. Central-stenosis, or lateral recess-stenosis.
Able to give informed consent and to answer the questionnaires.
Over 18 years of age
Able to understand Norwegian, both spoken and written
**Exclusion criteria:**
Degenerative lumbar spondylolisthesis, with a slip ≥ 3 mm verified on standing plain x-rays in lateral view.
Not willing to participate in the trial.
Former surgery at the level of stenosis
Fracture, or former fusion in the thoraco-lumbar region.
Cauda equina syndrome (bowel or bladder dysfunction) or fixed complete motor deficit
ASA-classified 4 or 5.
Over 80 years of age
Lumbosacral scoliosis >20°verified on AP-view
Distinct symptoms in one or both of their legs due to other diseases, e.g. polynevropathy, vascular claudication or osteoarthtritis.
Stenosis in>3e levels
Not able to comply fully with the protocol, including treatment, follow-up or study procedures (psychosocially, mentally and physical).
Participating in another clinical trial that may interfere with the present trial

© 2022 Hermansen E et al. *JAMA Network Open.*

eTable 2. Primary Outcome

Primary outcome was change of Oswestry Disability Index (ODI) after 3, 12 and 24 months. The proportion of success patient (success= 30% reduction in baseline ODI) in each group was also calculated. Corresponding 95% CI are given in parentheses. Independent samples t-test was used to test differences in mean change between two groups, and chi-square test was used to test differences in proportions between two groups.

	**Unilateral laminotomy with crossover** (UL)	**Bilateral laminotomy** (BL)	**Spinoous process osteotomy** (SPO)	**P-value (UL vs BL)**	**P-value (UL vs SPO)**	**P-value (BL vs SPO)**
Baseline (SD)	38.5 (14.9)	40.2 (14.1)	36.6 (14.3)	0.353	0.275	0.04
Change after 3 months (95% CI)	-20.6 (-23.0 - -18.2)	-21.2 (-23.9 - -18.4)	-17.7 (-20.1 - -15.2)	0.770	0.089	0.059
Change after 12 months (95% CI)	-19.1 (-22.1 - -16.1)	-20.0 (-22.7 - -17.2)	-17.8 (-20.7 - -14.9)	0.680	0.536	0.292
Change after 24 months (95% CI)	-17.9 (-20.8 - -14.9)	-19.7 (-22.7 - -16.8)	-19.9 (-22.8 - -17.0)	0.374	0.338	0.947
Proportion of success after 3 months (%-95% CI)	75.9 (67.8 – 82.9)	72.9 (64.5 – 80.3)	70.8 (62.4 – 78.3)	0.574	0.340	0.697
Proportion of success after 12 months (%-95% CI)	74.2 (65.9 – 81.5)	68.0 (59.1 – 75.9)	72.9 (64.5 – 80.3)	0.264	0.809	0.379
Proportion of success after 24 months (%, 96% CI)	67.4 (58.8 – 75.2)	67.5 (58.5 – 75.5)	73.5 (65.1 – 80.8)	0.993	0.277	0.289

© 2022 Hermansen E et al. *JAMA Network Open.*

eTable 3. Secondary Outcome

Secondary outcome was change in EQ-5D, ZCQ-score, NRS-score for back-pain, NRS-score for leg-pain and GPE-score at 3, 12 and 24 months All values are given as mean change from baseline to follow up except for GPE-score which is given as mean absolute score at each follow-up time. Corresponding 95% CI in parenthesis, and p-values from standard ANOVA test.

	Follow up after 3 months				Follow up after 12 months				Follow up after 24 months			
	**UL**	**BL**	**SPO**	**P-value**	**UL**	**BL**	**SPO**	**P-value**	**UL**	**BL**	**SPO**	**P-value**
**EQ-5D (95% CI)**	0.22 (0.18-0.26)	0.24 (0.20-0.27)	0.19 (0.15-0.23)	0.22	0.18 (0.13-0.22)	0.22 (0.18-0.26)	0.20 (0.16-0.23)	0.299	0.21 (0.16-0.25)	0.21 (0.17-0.26)	0.22 (0.18-0.25)	0.925
**ZCQ-symptoms (SD)**	-1.09 (-1.22--0.97)	-1.13 (-1.26--0.99)	-1.01 (-1.15--0.87)	0.46	-0.98 (-1.12--0.84)	-1.01 (-1.17--0.86)	-1.01 (-1.16--0.86)	0.948	-0.96 (-1.10--0.83)	-1.04 (-1.19--0.90)	-1.07 (-1.22--0.92)	0.569
**ZCQ-function (SD)**	-0.83 (-0.94--0.73)	-0.90 (-1.00--0.79)	-0.81 (-0.94--0.69)	0.57	-0.83 (-0.95--0.71)	-0.84 (-0.95--0.72)	-0.82 (-0.94--0.70)	0.978	-0.79 (-0.90--0.68)	-0.87 (-0.99--0.76)	-0.89 (-1.00--0.76)	0.458
**NRS Back-pain (SD)**	-2.99 (-3.46--2.53)	-3.16 (-3.66--2.65)	-2.51 (-2.94--2.08)	0.13	-2.60 (-3.11--2.09)	-2.82 (-3.32--2.32)	-2.80 (-3.23--2.37)	0.778	-2.64 (-3.17--2.12)	-2.52 (-3.09--1.96)	-2.81 (-3.24--2.37)	0.741
**NRS Leg-pain (SD)**	-3.64 (-4.12--3.16)	-3.72 (-4.27--3.17)	-3.41 (-3.93--2.89)	0.68	-3.02 (.84-2-3.56-	-3.27 (-3.82--2.73)	-3.40 (-3.95--2.84)	0.604	-3.30 (-3.83--2.78)	-3.63 (-4.14--3.12)	-3.59 (-4.15--3.03)	0.645
**GPE-score**	2.42 (2.23-2.61)	2.27 (2.11-2.43)	2.40 (2.18-2.62)	0.49	2.43 (2.21-2.64)	2.51 (2.28-2.74)	2.36 (2.13-2.59)	0.663	2.55 (2.30-2.79)	2.55 (2.32-2.78)	2.29 (2.06-2.53)	0.214

© 2022 Hermansen E et al. *JAMA Network Open.*

eTable 4. Reoperations During the Hospital Stay

	UL	BI	SPO	Total
**Postoperative hematoma**	1	1	2	4

© 2022 Hermansen E et al. *JAMA Network Open.*

eTable 5. Reoperation Recorded After Discharge and Until 3 Months of Follow Up

	UL	BL	SPO	Total
**Deep infection**	1	0	0	1
**Hematoma**	2	0	0	2
**Superficial infection**	0	1	0	1
**Disc Hernia**	0	1	0	1
**Reoperated right level**	1			1
**Sum**	4	2	0	6

© 2022 Hermansen E et al. *JAMA Network Open.*

eTable 6. Reoperation Recorded After 3 Months and Until 24 Months of Follow Up

	UL	BL	SPO	Total
**Disc Hernia**	2	1	2	5
**Spondylolisthesis**	2	0	3	5
**Re-decompression same level**	2	2	0	4
**Laminectomi**	0	0	1	1
**Reoperated different level**	2	0	1	3
**Sum**	8	3	7	18

© 2022 Hermansen E et al. *JAMA Network Open.*

eTable 7. Interactions

	UL	BL	SPO	Test for interaction effect	N eligible for analyses
**Change in inclusion criteria**					
Included before Nov 1st 2015	-19.8 (-23.4, -16.3)	-17.4 (-21.1, -13.6)	-20.1 (-23.9, -16.3)		
Included from Nov 1st 2015	-15.8 (-19.4, -12.2)	-19.9 (-23.6, -16.3)	-21.7 (-25.1, -18.2)	0.156	393
**Level of surgery**					
L2L3	-15.4 (-24.9, -5.9)	-21.1 (-28.1, -14.2)	-26.4 (-34.1, -18.7)		
L3L4	-17.5 (-21.2, -13.7)	-18.8 (-22.7, -14.9)	-19.9 (-23.9, -16.0)		
L4L5	-17.9 (-21.1, -14.6)	-18.0 (-21.1, -14.8)	-20.0 (-23.3, -16.7)	0.612	379
**Number of levels operated**					
1	-18.6 (-21.9, -15.2)	-18.8 (-22.2, -15.4)	-21.1 (-24.4, -17.8)		
2	-17.4 (-21.5, -15.2)	-17.5 (-21.8, -13.1)	-20.3 (-24.7, -15.9)		
3	-21.1 (-50.3, 8.1)	-27.1 (-40.2, -13.9)	-24.4 (-39.0, -9.8)	0.988	379

Means and corresponding 95% CI calculated by predicting marginal effects after fitting multilevel linear models with random intercept for operating hospital and adjusting for baseline ODI score, as well as including an interaction term between study arm and the covariates identifying the relevant subgroups. Separate models were fitted to analyze each interaction of interest. P-values are calculated from a likelihood ratio test comparing models with and without the interaction term of interest.

© 2022 Hermansen E et al. *JAMA Network Open.*

eFigure 1. Secondary Outcomes After 3 Posterior Decompression Techniques for Lumbar Spinal Stenosis

Secondary outcomes after three posterior decompression techniques for lumbar spinal stenosis. Given with values from baseline and at follow up at 3, 12 and 24 months. EQ5D; EuroQol 5 Dimension questionnaire. ZCQ; Zurich Claudication Questionnaire, NRS; Numeric Rating Scale and GPE; Global Perceived Effect scale. UL: Unilateral Laminotomy with crossover, BL: Bilateral Laminotomy and SPO: Spinous Process Osteotomy.

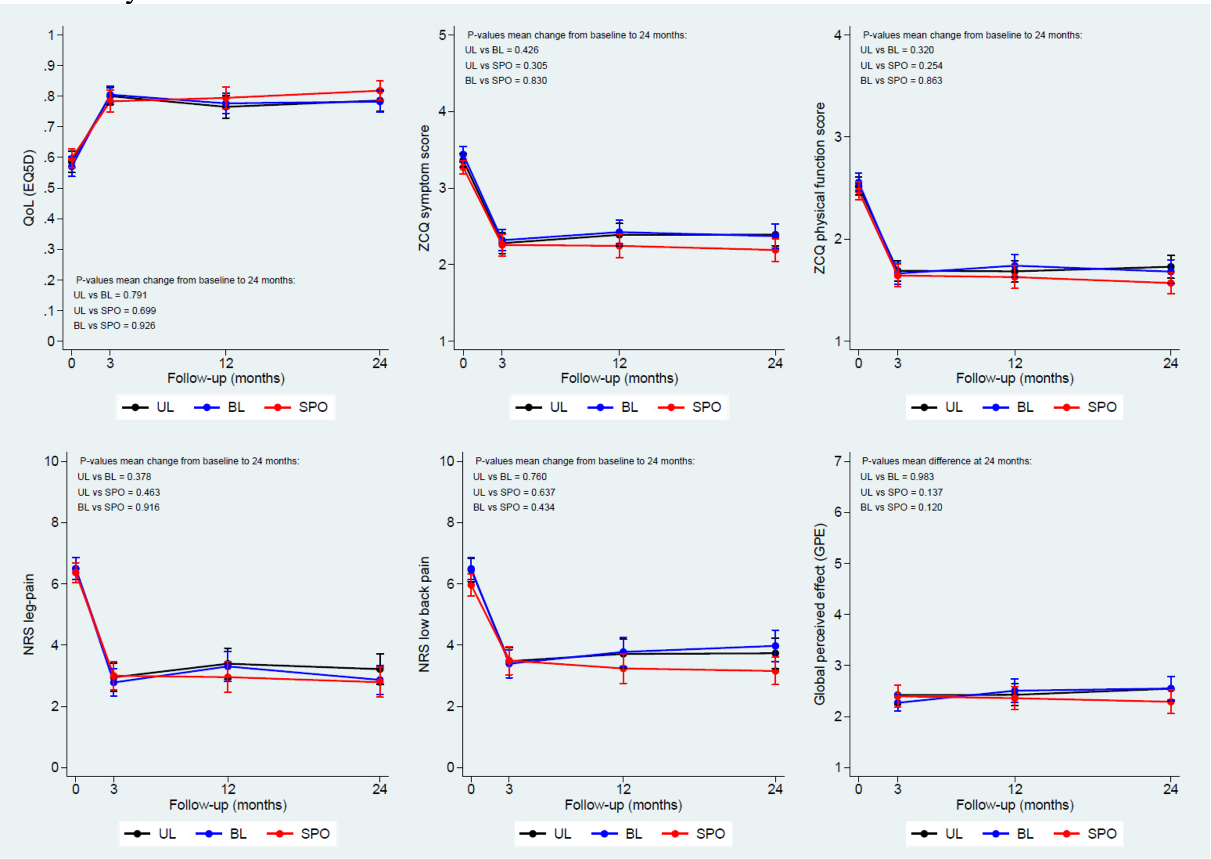

© 2022 Hermansen E et al. *JAMA Network Open.*

eFigure 2. Global Perceived Effect

Global Perceived Effect- scale given in a 7-point Likert scale. UL: Unilateral Laminotomy with crossover, BL: Bilateral Laminotomy and SPO: Spinous Process Ostetomy.

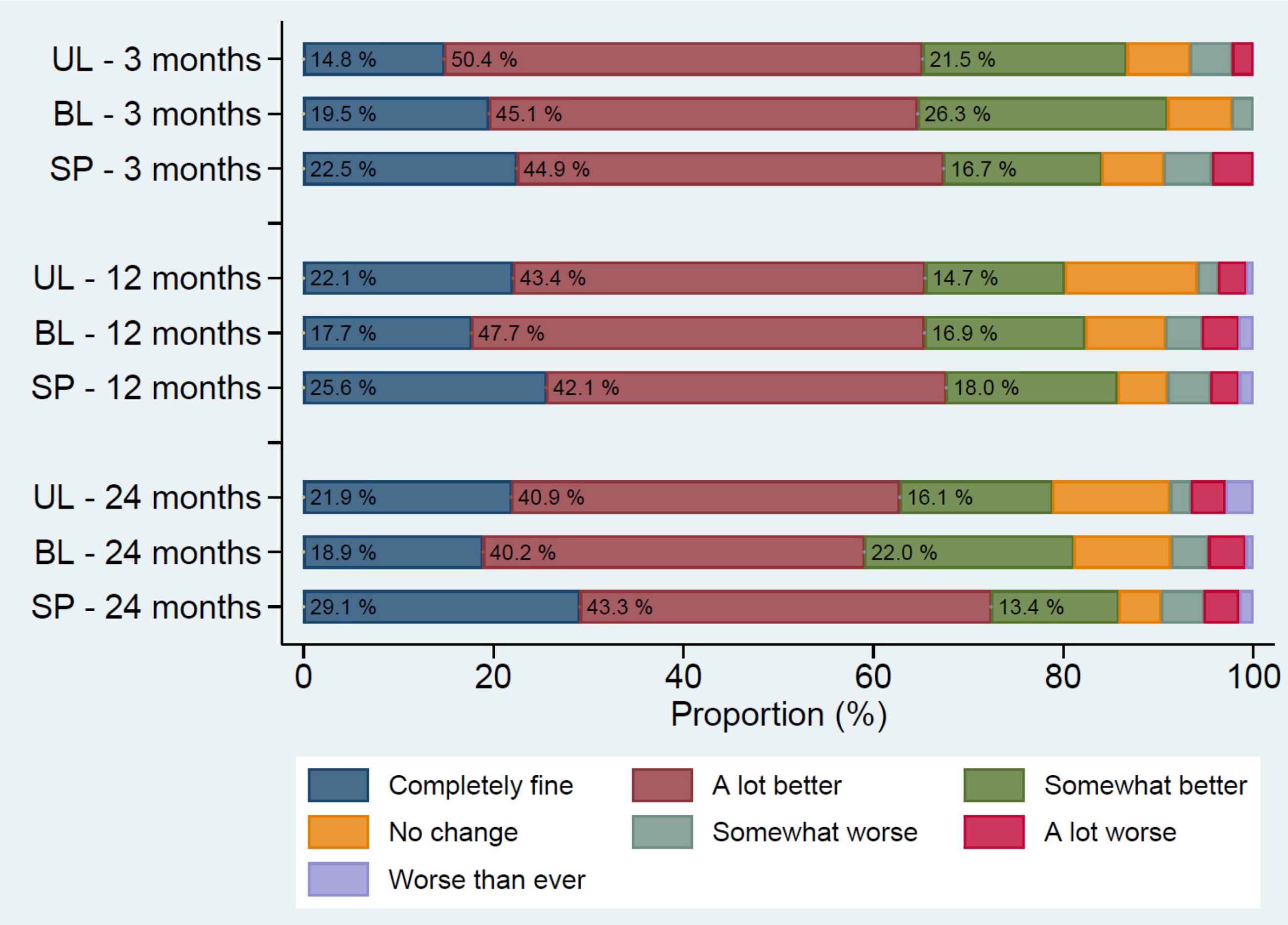

© 2022 Hermansen E et al. *JAMA Network Open.*

eFigure 3. Distribution of the Primary Outcome (Change in ODI) for Each Study Arm

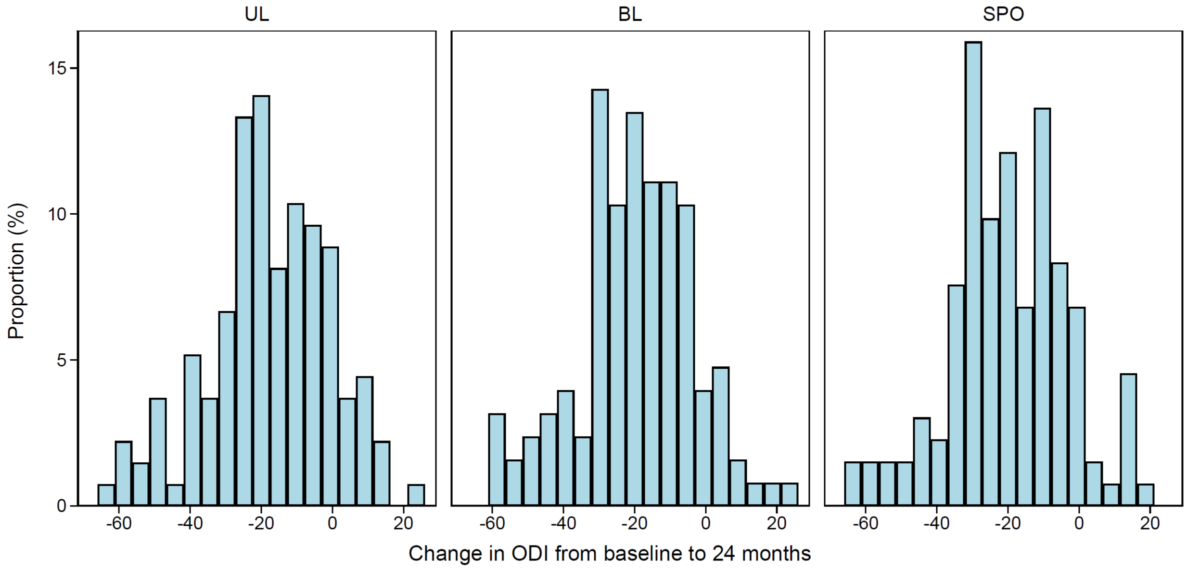

© 2022 Hermansen E et al. *JAMA Network Open.*

eFigure 4. Cumulative Number of Included Patients Over Time

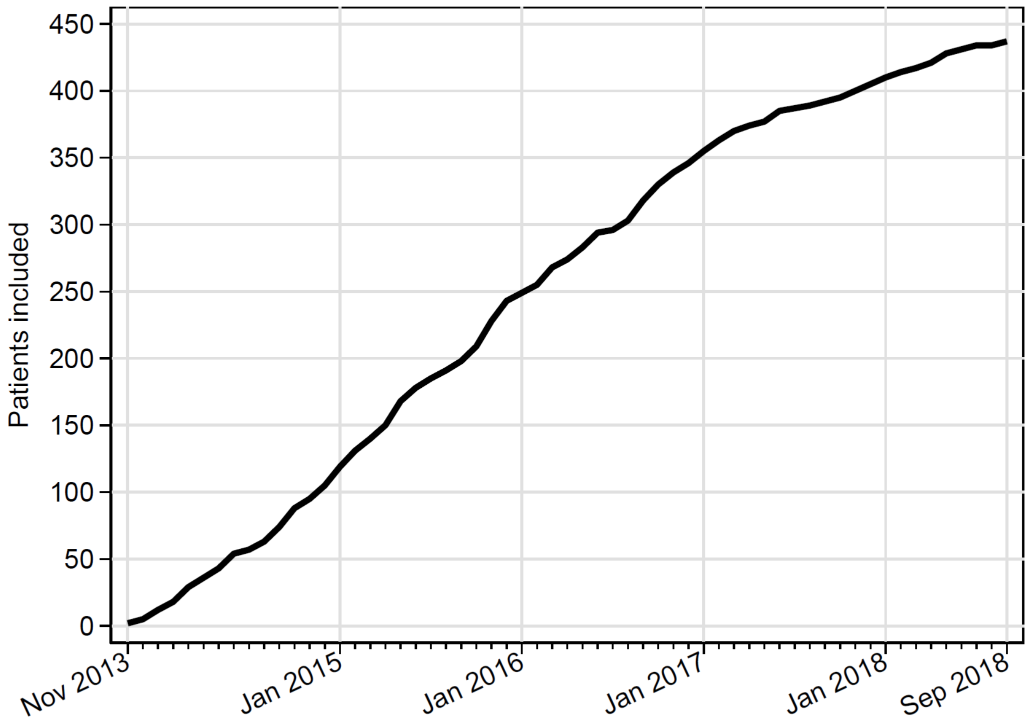

© 2022 Hermansen E et al. *JAMA Network Open.*

eAppendix 1. The NORDSTEN-SST Study Study Monitor Report

Written By Katarina Mølsæter, Special Research Advisor Clinical Trials, Møre and Romsdal Hospital Trust.

1.1 Background

Ethical obligations have left its mark over decades within human clinical trials. The Nuremberg Code in 1947 became the first document that referred to ethical regulation based on informed consent in connection with human trials [1, 2]. Further spin-off, the World Medical Association (WMA) published the Helsinki Declaration [3] in 1964, sets requirements for ethical principles in clinical research in humans [2, 4]. In the early 1960s, the Thalidomide disaster aroused the need for increased control of patient safety in clinical drug testing. International Conference on Harmonization of Good Clinical Practice Guidelines (ICH-GCP) is the final document and an extension of the Helsinki Declaration [2, 3, 5]. ICH-GCP refers to a standard for design, planning, implementation, monitoring, storage, and reporting of clinical studies of scientific quality. The accuracy and quality of the research data obtained and ensured in parallel with the fact that the integrity, rights and confidentiality of the participants are safeguarded [5-9].

Similar quality systems, such as in clinical drug studies, are incomplete in clinical orthopaedic intervention studies without drugs. Orthopaedic research desires an implementation of Good Clinical Practice (GCP) standard for randomized control trials to account scientific research quality, practical and ethical aspects [10, 11].

1.2 Modified model of GCP

Based on ICH-GCP [5] guidelines, a development of a modified model of GCP adapted The NORDSTEN-SST trial accomplished, though it is not required by law. Aspects and tasks related to drug testing was replaced with current study-specific routines such as operating method, examination and follow-up of the included patient. The NORDSTEN-SST trial has chosen to use a modified model of GCP to ensure research ethics for included patients, enhance quality of obtained data and to initiate good research structure. Prior start-up, an external monitor [5] (in person and not involved in recruitment and data collection) in collaboration with sponsor, have determined a monitor plan. This plan was developed primary to identify transcript errors, regulatory administration, informed consent process and study related tasks. Source data verification (SDV) is by definition the process of ensuring the reflection of data accuracy at the clinical site [12]. Based on this assessment, a risk-based monitoring plan addition covering endpoints, received operative treatment according to randomization, respect to patients safety and integrity mirror the research protocol.

Organized study team consisting study coordinator and responsible orthopaedic principal investigators (PI) at each participating hospitals [13]. A well-functioned supportive administrative team attend to recruitment process as an ethical responsibility protecting the patient's rights [14, 15], and at the same time ensure the research project progress in accordance with the research protocol.

© 2022 Hermansen E et al. *JAMA Network Open.*

The modified model of GCP elected into three main stages; planning, implementation and termination, were last stage includes archiving and transmission of obtained data. Stages has been adapted to secure accountability for transparency during implementation.
Planning Template for research –and surgical protocols ensured participating hospitals identical procedures during implementation of the clinical trial, including pre-assessments prior randomized surgical technique. Checklist for participant to maintain accurate achievement, follow-up and case report form (CRF) [16] reflecting the research protocol and endpoints for the trial designed. To evaluate safety and serious incident, CRF for serious adverse events (SAE) [5] formed. Study folder contain essential documents and signed informed consent established in an adapted version of Investigator Site file (ISF), located at participating hospital, and Trial Master file (TMF) located at sponsor. This to conduct research structure for administrative clarity and ethical obligations. The Health and Research Act [7] regulates the consent process but is vague in relation to the practical implementation. ICH-GCP [5] on the other hand has a more detailed description of the process and therefore an adaption to secure ethical principals. Prior start-up, monitor initiated specific training of ethical consent process in the same process as introduction of the modified model in consultation with trial staff.
Implementation Screening and inclusion of participant according to selection criteria, the informed consent process has been carried out in accordance with legislation [7], where orthopaedic or study coordinator with relevant training obtained the consent. CRF continuously filled out by study coordinator or PI with patient medical record or consultation as source data. Completed CRF continued transferred to authorized electronic database. Evaluation of safety and well-being at participating patients frequently assessed. This considering determining surgical effect and extending attendance. During active phase of the trial, storage of identified information placed separate with restricted access and separate from CRF.
Termination The NORDSTEN-SST trial has not reached study termination and this stage indicate approach actions. Long-term archiving of essential documents (ISF and TMF), coded CRF and signed informed consent will be stored separate at suitable location at participated hospitals. A copy of CRF will be sent to the Faculty of Research support, University of Oslo for archiving. Archived patient data (including deidentified data and informed consent) stored in set period as stated in the Regional Ethics Committee approval. In accordance with regulated law, patient data attend to be destroyed or pseudonymized after declared date.

1.3 Monitor plan and deviation

The monitor plan pays special attention to participant’s rights and safety in the study. Further, overseeing the progress of the study and ensure the study is conducted, recorded, and reported in accordance with the protocol and the applicable regulatory requirements. Participating hospitals have annually been visited and monitored according to set monitor plan and SDV.

- Informed consent 100% of participated patients
- Selections criteria 10% of participated patients
- 100% of participated patients at time period randomization to 2 years follow-up

© 2022 Hermansen E et al. *JAMA Network Open.*

- Serious adverse event
  - Randomization and retrieved operative treatment
  - Radiological examination (MRI and x-ray), pre and post-operative treatment at 3 month and 2 years.
  - Clinical visit at baseline, 3 months and 2 years.
- Revised and adapted table of content for study folder ISF and TMF checked for essential documents, delegation log and curriculum vitae (CV) for study team, archiving of signed informed consent.

1.4 Classification and recorded deviations

Deviations are common in clinical trials and varies in their incidence and impact. Classification of deviation can be processed different but the area of patient ethical and safety, and data quality are crucial. Ethical concerns should only be made with consent of the regulatory authorities and Institutional Ethics Committees (ECs), and deviation hence unethical. The impact on data quality needs to be analysed, because the data is the most important outcome of the trial [17]. The classification of deviations is categorized according to impact on data quality and safety. The protocol, among other things, reflects a tight time window for examinations but they were performed prematurely or within delayed time. Post-surgery, adverse events are within surgical treatment occasionally expected. But due to implementation of GCP, as seen in table 1 and classification grade 4, set deviations according to research protocol are stated.

According to statistical considerations in Statistical Analysis Plan (SAP) for NORDSTEN-SST Version 4.2, definitions of major deviations from protocol represents Grade 4.

Recorded deviations at monitoring procedure are representing the time-period at post-operative surgery at hospitalization, 3 month and 2-year post-operative follow-up. The deviations are a total of 18 participating hospitals, 437 included participating patients.

Classification of deviations

- Grade 1: No impact on data quality or patient safety
- Grade 2: Minor impact on data quality
- Grade 3: Minor impact on patient safety
- Grade 4: Major impact on data quality or patient safety
- Grade 5: Participating leading to patient serious adverse event and/or death

© 2022 Hermansen E et al. *JAMA Network Open.*

Classification	Deviation	Number of deviations
**Grade 1**	○ Incomplete delegation log and CV, copy of regulative approval (per included centre)	○ 14
**Grade 2**	○ Radiological examination post-operative treatment taken out of time range<br>○ Clinical visit post-operative treatment out of time range	○ 101<br>○ 63
**Grade 3**	○ Incomplete signed informed consent process as; missing date/signature from either patient/orthopaedic/study coordinator, patient/orthopaedic/study coordinator signed post randomization, wrong informed consent signed	○ 35
**Grade 4**	○ Not received operative treatment in accordance with randomized allocation<br>○ Received operative treatment in accordance with randomized allocation and operated with a new operation at same level during the follow-up period<br>○ Not provided informed consent<br>○ Withdrew the informed consent and claimed their data withdrawn from analyses<br>○ Post-operative treatment leading to adverse event (AE) Post-operative/ 3month control (see table 3)	○ 18<br>○ 3<br>○ 0<br>○ 0<br><br>○ 58/76
**Grade 5**	○ Post-operative treatment leading to serious adverse event (SAE)	○ 3

1.5 Evaluation of modified model of GCP and cooperation with study coordinators

Yearly monitor visit was completed in close dialogue and collaboration with study coordinator at participating hospitals. Electronic medical journal, signed informed consent, and CRFs were used as source of data verifications. Detected deviations were consequent recorded as Note to

© 2022 Hermansen E et al. *JAMA Network Open.*

file-form. Note til file errors reflecting misspelling in CRFs were corrected at local hospital and conveyed adjustment in electronic database at Faculty of Research support, University of Oslo. Copy of note to file recorded at included hospital were sent to Faculty of Research support, University of Oslo as a part of sponsors responsibility of detection and handling deviations.

In the starting phase of the trial, early identified note to file led to an opportunity for monitor and study coordinator to solve the possible challenge that forced deviations. Proper and increased approximation to the trial showed results of less note to file and expand interests from study coordinator further in the study. Study coordinator responded in dialogue with monitor the importance of being a key person to deliver high quality research data [18]. As a result of the implementation of the modified model of GCP, the study coordinator experienced a common understanding with the purpose of new guidelines and a confidence in the monitor emerged. Considering the work on updating guidelines, monitoring can affect data validity and facilitate implementation.

1.6

1.7 Additional information

Author of Supplementary, Katarina Mølsæter, was monitor in the NORDSTEN-SST study. In December 2018, she defended her master's theses at the Faculty of Medicine and Health Science at Norwegian University of Science and Technology (NTNU). The master theses were performed with qualitative research design with individual depth interviews with study coordinators participating in the NORDSTEN-SST study. The article (not yet published) has the title Study coordinators experience with the use of a modified model of Good Clinical Practice (GCP) in orthopaedic research in Norway.

Adverse event (AE)	Any untoward medical/surgical/neurological occurrence in a patient or clinical investigation subject undergoing operative treatment and which does not necessarily have a causal relationship with this treatment. An adverse event can therefore be any unfavourable and unintended sign (including an abnormal laboratory)
Investigator Site file (ISF)	Contains the essential documents to be archived by the Principal Investigators (PI).<br>The Sponsor is responsible for providing the PI with an ISF, and the PI is responsible for creating, storing and updating the essential documents throughout the study.
Monitor	An independent person without influence in scientific work and performing data collection
Monitoring	The act of overseeing the progress of a clinical trial, and of ensuring that it is conducted, recorded, and reported in accordance with the protocol, Standard Operating Procedures (SOPs), modified model of Good Clinical Practice, and the applicable regulatory requirement(s).<br><br>The purposes of trial monitoring are:<br>-to verify that the rights and well-being of human subjects are protected and<br>-the reported trial data are accurate, complete, and verifiable from source documents.
Principal Investigator (PI)	A person responsible for the conduct of the clinical trial at a trial site. If a trial is conducted by a team of individuals at a trial site, the investigator is the responsible leader of the team and may be called the principal investigator.
Serious Adverse Event (SAE)	Any untoward medical occurrence that at any dose:<br>- results in death,

© 2022 Hermansen E et al. *JAMA Network Open.*

	- is life-threatening,<br>- requires inpatient hospitalization or prolongation of existing hospitalization,<br>- results in persistent or significant disability/incapacity, or<br>- is a congenital anomaly/birth defect
Sponsor	An individual, company, institution or organization, which takes responsibility for the initiation, management, and/or financing of a clinical trial.
Trial Master file (TMF)	Contains the essential documents to be archived by Sponsor, and these documents are owned by Sponsor. It is Sponsor's responsibility to create, store and update the TMF. These tasks can be delegated. If so, delegation of tasks must be documented.

© 2022 Hermansen E et al. *JAMA Network Open.*

eAppendix 2. The NORDSTEN Collaboration

The NORDSTEN collaboration is responsible for three ongoing studies. In addition to the present trial (the NORDSTEN-SST), the NORDSTEN- Degenerative Spondylolisthesis-trial (NORDSTEN-DS) trial is an RCT investigating whether "Decompression Alone" is non-inferior to "Decompression and Fusion" for lumbar spinal stenosis with spondylolisthesis. The third of the three NORDSTEN trials is the NORDSTEN-Observational Cohort (NORDSTEN-OC). This study is a prospective longitudinal observational study investigating the natural course of patients with spinal stenosis not opted for surgical treatment.
For the surgical studies, the primary endpoint is 2 year after inclusion. Long-term results will be evaluated at 5 and 10 years. So far, five Ph.D. candidates have been affiliated with the NORDSTEN studies, of which two of them have completed the Ph.D. grade.

The organization

The NORDSTEN collaboration is organized in a Scientific Board, an Administrative Executive Board, a Study Coordinator Centre, a Data safety/Monitory Board and a Working Committee.
Scientific Board:
This group have the scientific responsibility for study. This means that this group have the responsibility for writing the scientific protocols and the scientific reviews.
Leader: Christian Hellum, MD PhD.
Members: Kjersti Storheim PT Phd, Kari Indrekvam MD PhD, Jens Ivar Brox MD PhD, Oliver Grundnes MD PhD, Tore Solberg MD PhD, Ivar Magne Austevoll MD PhD, Erland Hermansen MD PhD, Frode Rekeland MD PhD candidate, Jørn Aaen MD PhD Candidate, Clemens Weber MD PhD, Eric Franssen MD, Hasan Banitalebi MD PhD Candidate and Masoud Anwar MD PhD.
International Collaborator: Prof Helena Brisby MD PhD (Sweeden).
Patient representative: Inger Ljøstad. affiliated to The Norwegian back and spine association (Ryggforeningen in Norway), a membership organization which mainly comprises people suffering from back pain.

Administrative Executive Board:
This group have the administrative and economic responsibility for the study.
Leader: Kari Indrekvam MD PhD.
Members: Anniken Remseth, Department of Research, Møre and Romsdal Hospital Trust, Kjersti Storheim (FORMI), Christian Hellum, Erland Hermansen, Frode Rekeland and Ivar M Austevoll.

© 2022 Hermansen E et al. *JAMA Network Open.*

Responsibility for the different NORDSTEN-study cohorts:
SST: Erland Hermansen.
DST: Ivar Magne Austevoll.
OC: Frode Rekeland.

Study Coordinating Centre:
FORMI (Research Unit for musculoskeletal research at Oslo University Hospital) is the study coordinating centre. They are responsible for nationwide coordination and communication with the participating hospitals and ensures that all data are collected and stored according to the study protocol.
Leader: Kjersti Storheim
Data safety/Monitory Board:
The Clinical Trial Unit at Oslo University Hospital is responsible for data storage and safety. Katarina Mølsæter is responsible for monitoring of the study. This is done after a modified version of the ICH-GCP.

The NORDSTEN-SST Working group:

Akershus University Hospital, Orthopedic department:
Oliver Grundnes, Therese Gundersen and Trine Myrvold.
Sørlandet Hospital Trust, Arendal Hospital, Orthopedic department:
Yngve Sporstøl, Ted P. Lundgren, Anne-Charlotte Fosse Haug, Elisabeth Lilleholdt Muller
Sørlandet Hospital Trust, Kristiansand Hospital, Orthopedic department:
Odd Arild Ågedal, Kjartan Krogedal and Bettina Timenes
Vestre Viken Hospital Trust, Bærum Hospital, Orthopedic department.:
Vidar Opland and Merete Finjarn
Innlandet Hospital Trust, Division Elverum, Orthopedic department:
Arnfinn Pedersen and Hege Westgård.
Innlandet Hospital Trust, Division Gjøvik, Orthopedic department:
Håvard Furunes and Hege Bergum Nilsen
Vestre Viken Hospital Trust, Drammen Hospital, Orthopedic department:
Lars Hubshle
Haukeland University Hospital, Orthopedic department:
Maria Rieber-Mohn and Andreas Seip,

© 2022 Hermansen E et al. *JAMA Network Open.*

Haukeland University Hospital, Ortopedic department., Kysthospitalet i Hagevik:
Turid Rognsvåg and Janne Haugland.
Innlandet Hospital Trust, Division Lillehammer, Orthopedic department:
Greger Lønne and Gisle Szacinski
Oslo University Hospital, Ullevål, Ort. Dept. and Division of Clinical Neuroscience:
Espen Sjåberg and Ellen Aksnes.
Stavanger University Hospital, Orthopedic department and Neurosurgical department:
Eric Franssen and Kirstine Eikenes.
Martina Hansens Hospital:
Nikolaos Ikonomou and Ove Bjørnstad
University Hospital of Northern Norway, Neurosurgical department:
Andreas Sørlie and Kristine Evanger,
Telemark Hospital Trust, Skien Hospital, Orthopedic department
Knut Jørgen Haug and Ingrid Edborg
Møre and Romsdal Hospital trust, Ålesund Hospital, Orthopedic department:
Jørn Aaen, Vidar Punsvik and Anita Karin Vassbakk.
Northern Trøndelag Hospital trust, Levanger Hospital, Orthopedic department:
Arild Hjulstad, Leif Kipsgård, Grete Ward and Turid Fjesme

© 2022 Hermansen E et al. *JAMA Network Open.*

Participating hospitals with numbers of patients included

Hospital	Number of patients
Akershus University Hospital, Orthopedic department	24
Sørlandet Hospital Trust, Arendal Hospital, Orthopedic department	21
Sørlandet Hospital Trust, Kristiansand Hospital, Orthopedic department	5
Vestre Viken Hospital Trust, Bærum Hospital, Orthopedic department	28
Vestre Viken Hospital Trust, Drammen Hospital, Orthopedic department	3
Innlandet Hospital Trust, Division Elverum, Orthopedic department	1
Innlandet Hospital Trust, Division Gjøvik, Orthopedic department	39
Haukeland University Hospital, Orthopedic department	4
Haukeland University Hospital, Kysthospitalet i Hagevik Orthopedic department	62
Innlandet Hospital Trust, Division Lillehammer, Orthopedic department	15
Oslo University Hospital, Ullevål, Orthopedic department.	36
Stavanger University Hospital, Orthopedic department and Neurosurgical department	57
Martina Hansens Hospital	30

© 2022 Hermansen E et al. *JAMA Network Open.*

University Hospital of Northern Norway, Neurosurgical department.	6
Telemark Hospital Trust, Skien Hospital, Orthopedic department.	17
Møre and Romsdal Hospital trust, Ålesund Hospital, Orthopedic department	61
Northern Trøndelag Hospital trust, Levanger Hospital, Orthopedic department	28
**Total**	**437**

Organization chart for the NORDSTEN-study:

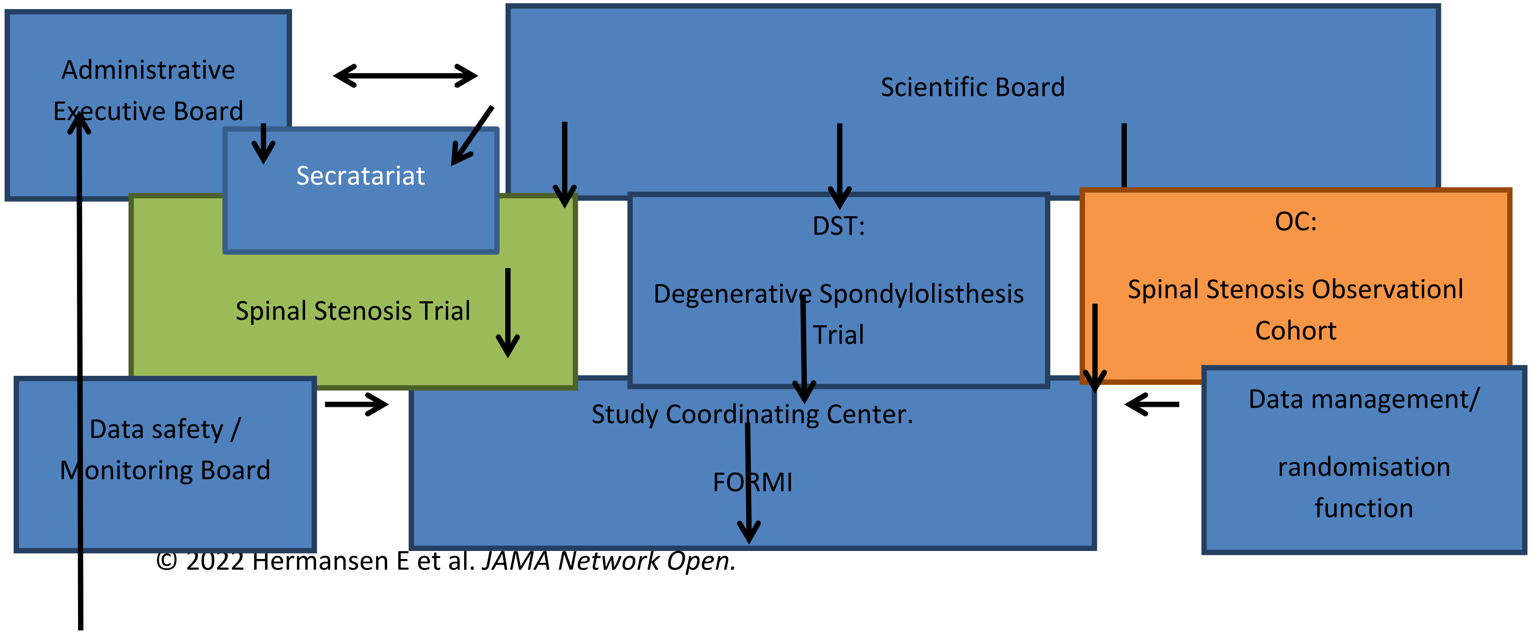

© 2022 Hermansen E et al. *JAMA Network Open.*

Working Commitee.

Participating hospitals

© 2022 Hermansen E et al. *JAMA Network Open.*

eReferences.

1. Vollmann, J. and R. Winau, *Informed Consent In Human Experimentation Before The Nuremberg Code.* BMJ: British Medical Journal, 1996. **313**(7070): p. 1445-1447.
2. Brearley, S., *How should we conduct our research in line with medical research regulations?* Journal of Diabetes Nursing, 2013. **17**(3): p. 116-117.
3. Word Medical Association, *World Medical Association Declaration of Helsinki: ethical principles for medical research involving human subjects.* JAMA, 2013. **310**(20): p. 2191.
4. Bhandari, M. and A. Joensson, *Clinical research for surgeons*. 2009, Stuttgart: Thieme.
5. ICH. *ICH Harmonised Guideline. Intergrated Addendum to ICH E6(R1): Guideline for Good Clinical Practice E6(R2). Current step 4 version*. 2016 [cited 2017 20. September]; Available from: https://www.ich.org/fileadmin/Public_Web_Site/ICH_Products/Guidelines/Efficacy/E6/E6_R2__Step_4_2016_1109.pdf.
6. Wagner-Bohn, A., et al., *Implementing good clinical practice in two noncommercial phase II studies in children with cancer.* Oncology Research and Treatment, 2007. **30**(1-2): p. 21-26.
7. Services, M.o.H.a.C., *The Health and Research Act 2008-06-20 nr 44*. 2008.
8. Education, M.o., *Act on the organization of research ethics work 2017-04-28*. 2017.
9. Services, M.o.H.a.C., *Regulations on clinical trials of medicinal products for human use FOR-2009-10-30 nr 1321*. 2009.
10. Mithoefer, K., et al., *Guidelines for the Design and Conduct of Clinical Studies in Knee Articular Cartilage Repair: International Cartilage Repair Society Recommendations Based on Current Scientific Evidence and Standards of Clinical Care.* Cartilage, 2011. **2**(2): p. 100-21.
11. Industry, G.f., *Guidance for Industry Preparation of IDEs and INDs for Products Intended to Repair or Replace Knee Cartilage*. 2011, Food and Drug Administration (FDA).
12. Sheetz, N., et al., *Evaluating Source Data Verification as a Quality Control Measure in Clinical Trials.* Ther Innov Regul Sci, 2014. **48**(6): p. 671-680.
13. Horwood, J., E. Johnson, and R. Gooberman-Hill, *Understanding involvement in surgical orthopaedic randomized controlled trials: A qualitative study of patient and health professional views and experiences.* International journal of orthopaedic and trauma nursing, 2016. **20**: p. 3-12.
14. Bowrey, S. and J.P. Thompson, *Nursing research: ethics, consent and good practice.(Report).* Nursing Times, 2014. **110**(1-3): p. 20-23.
15. Halladin Rauh, K., S. Hedegaard, and J. Rosenberg, *The ethical dilemmas when including patients in clinical trials.* Nordic Journal of Nursing Research, 2015. **35**(3): p. 133-135.
16. Bellary, S., B. Krishnankutty, and M. Latha, *Basics of case report form designing in clinical research.(Clinical Data Management)(Report).* Perspectives in Clinical Research, 2014. **5**(4): p. 159.

© 2022 Hermansen E et al. *JAMA Network Open.*

17. Ghooi, R.B., et al., *Assessment and classification of protocol deviations.* Perspect Clin Res, 2016. **7**(3): p. 132-6.
18. Connolly, B., et al., *Characterising the research profile of the critical care physiotherapy workforce and engagement with critical care research: a UK national survey.* BMJ Open, 2018. **8**(6).

© 2022 Hermansen E et al. *JAMA Network Open.*
