# Supplementary material for: Comparison of 3 Different Minimally Invasive Surgical Techniques for Lumbar Spinal Stenosis: A Randomized Clinical Trial
Source: JAMA Netw Open. 2022 Mar 28;5(3):e224291. doi: 10.1001/jamanetworkopen.2022.4291 (PMC8961320; doi:10.1001/jamanetworkopen.2022.4291)
Supplement: Supplement 3. — Data Sharing Statement [file jamanetwopen-e224291-s003.pdf]

Data Sharing Statement

Hermansen. Comparison of 3 Different Minimally Invasive Surgical Techniques for Lumbar Spinal Stenosis. *JAMA Netw Open*. Published March 28, 2022.
doi:10.1001/jamanetworkopen.2022.4291

Data
Data available: Yes
Data types: Deidentified participant data
How to access data: After submitting a written request to the scientific steering committee, data will be available upon reasonable request
When available: With publication

Supporting Documents
Document types: Statistical/analytic code, Informed consent form
How to access documents: There is already a SAP attached to the submission. The ICF-form is also available upon request
When available: With publication

Additional Information
Who can access the data: After submitting a written request to the scientific steering committee, data will be available upon reasonable request by an appropriate authority
Types of analyses: Upon reasonable request, deidentified data can be provided
Mechanisms of data availability: The request will go through an evaluation by the steering commitee before signing a data access agreement
